# Liquid-phase ASEM imaging of cellular and structural details in cartilage and bone formed during endochondral ossification: Keap1-deficient osteomalacia

**DOI:** 10.1038/s41598-021-84202-z

**Published:** 2021-03-11

**Authors:** Eiko Sakai, Mari Sato, Nassirhadjy Memtily, Takayuki Tsukuba, Chikara Sato

**Affiliations:** 1 Division of Dental Pharmacology, Department of Developmental and Reconstructive Medicine, Nagasaki University Graduate School of Biomedical Sciences, 1-7-1 Sakamoto, Nagasaki, 852-8588 Japan; 2 Health and Medical Research Institute, National Institute of Advanced Industrial Science and Technology (AIST), Central 6, Higashi 1-1-1, Tsukuba, Ibaraki 305-8566 Japan; 3 Traditional Uyghur Medicine Institute of Xinjiang Medical University, 393 Xinyi Rd, Urumqi, 830011 Xinjiang Uyghur Autonomous Region China

**Keywords:** Cartilage, Cartilage development, Bone, Biomaterials, Scanning electron microscopy

## Abstract

Chondrogenesis and angiogenesis drive endochondral ossification. Using the atmospheric scanning electron microscopy (ASEM) without decalcification and dehydration, we directly imaged angiogenesis-driven ossification at different developmental stages shortly after aldehyde fixation, using aqueous radical scavenger glucose solution to preserve water-rich structures. An embryonic day 15.5 mouse femur was fixed and stained with phosphotungstic acid (PTA), and blood vessel penetration into the hypertrophic chondrocyte zone was visualised. We observed a novel envelope between the perichondrium and proliferating chondrocytes, which was lined with spindle-shaped cells that could be borderline chondrocytes. At postnatal day (P)1, trabecular and cortical bone mineralisation was imaged without staining. Additional PTA staining visualised surrounding soft tissues; filamentous connections between osteoblast-like cells and osteocytes in cortical bone were interpreted as the osteocytic lacunar-canalicular system. By P10, resorption pits had formed on the tibial trabecular bone surface. The applicability of ASEM for pathological analysis was addressed using knockout mice of *Keap1*, an oxidative-stress sensor. In *Keap1*^−/−^ femurs, we observed impaired calcification and angiogenesis of epiphyseal cartilage, suggesting impaired bone development. Overall, the quick ASEM method we developed revealed mineralisation and new structures in wet bone tissue at EM resolution and can be used to study mineralisation-associated phenomena of any hydrated tissue.

## Introduction

**Figure 1:**
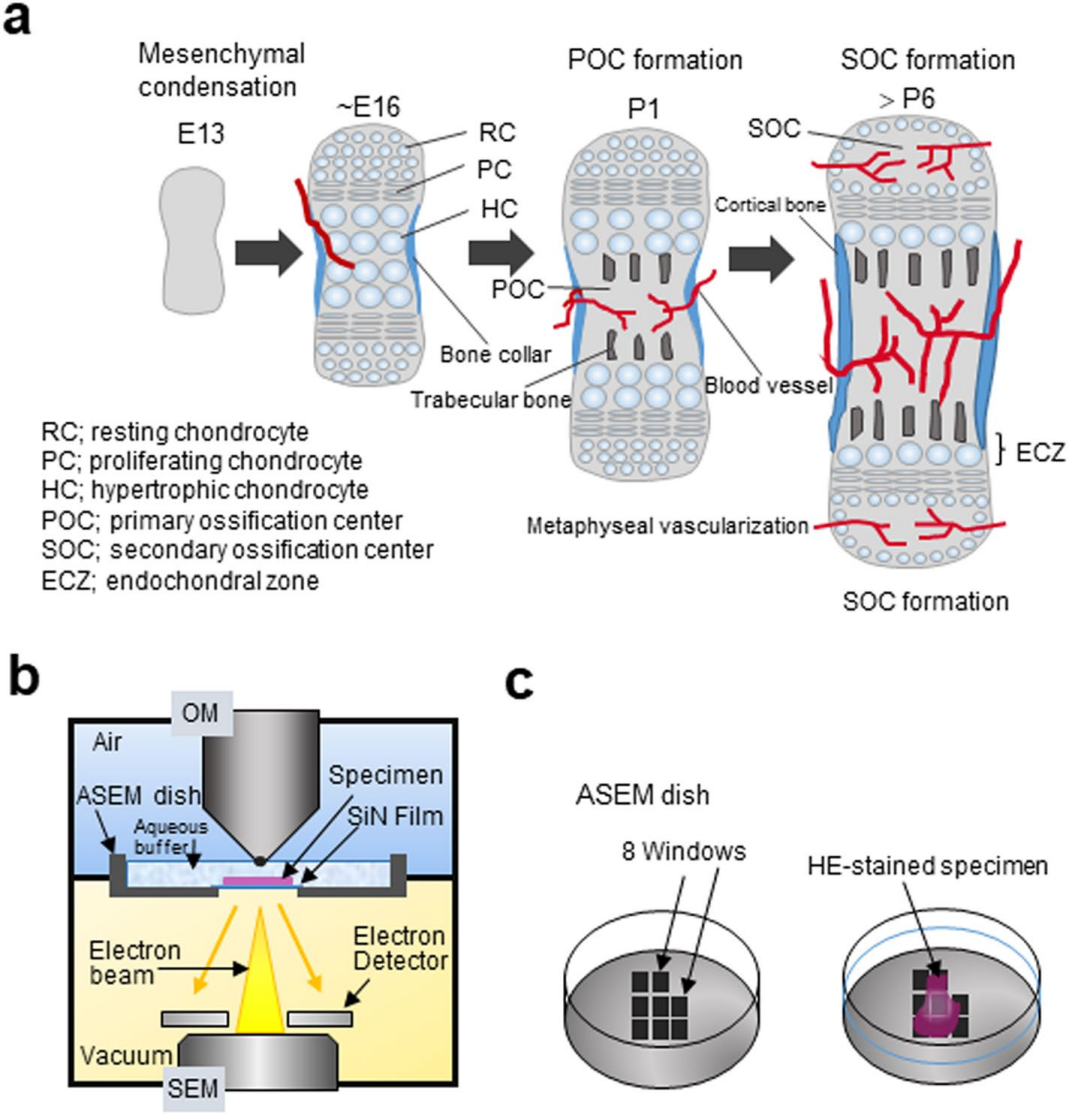
Schematic diagrams of chondrogenesis and endochondral ossification during bone development and ASEM methodology. (**a**) In ~ E13 mice, mesenchymal cells condense to form elongated rod shape structures and differentiate into chondrocytes at ~ E16. In the centre of the developing bone, chondrocytes become hypertrophic and induce the invasion of blood vessels. At P1, metaphyseal vascularisation is promoted, and a primary ossification centre is formed. From P6, blood vessels invade the epiphysis and contribute towards the formation of a secondary ossification centre. (**b**) The ASEM system used to image stained and unstained bone tissues. From below, an electron beam penetrates an SiN film window in the base plate of the ASEM dish and proceeds into the aldehyde-fixed specimen immersed in the buffer. Backscattered electrons are captured using a backscattered electron imaging detector. An optical microscope is positioned above the inverted SEM, with the specimen stage in between. (**c**) Schematic drawing of the removable 35-mm ASEM specimen dish and the eight SiN film windows in its bottom plate, which separate the atmosphere from the vacuum of the SEM column. Each window is 250 × 250 μm^2^ and the SiN film is 100 nm thick. HE-stained specimens are placed on the ASEM windows and immersed in radical scavenger buffer.

Most mammalian skeletal bones are classified as long bones, and development is initiated by mesenchymal cell condensation and subsequent chondrocyte differentiation^1,2^. Round chondrocytes in the resting chondrocyte zone (RCZ), differentiate into flat proliferating chondrocytes in the proliferating chondrocyte zone (PCZ), that later become hypertrophic chondrocytes in the hypertrophic chondrocyte zone (HCZ) (Fig. 1a). Terminal hypertrophic chondrocytes eventually die by apoptosis after the deposition of calcium phosphate. Thus, morphology of chondrocytes is closely related to the differentiation stage and function of the cells^3^. Recently, it was hypothesised that some chondrocytes escape apoptosis and survive to become osteoblasts. In vivo murine lineage-tracing studies demonstrated that some PTHrP^+^ chondrocytes could form osteoblasts through chondrocyte-to-osteoblast transformation^4^ and that chondrocyte-derived bone cells contribute to bone growth as well as remodelling^5^. Furthermore, using similar techniques, Mizuhashi et al. revealed that “borderline chondrocytes” vertically aligned with other chondrocytes peripheral of the growth plates become osteoblasts^6^. However, the origin of these unique chondrocytes is unknown, and it is unclear how they differentiate and how they move through the growth plate.

During angiogenesis, blood vessel penetration into the HCZ initiates bone formation^7^, as it allows progenitor cells that mature into osteoblasts and osteoclasts to move into the HCZ^8^. Bones are formed through the interactions between chondrocytes, osteoblasts, osteocytes, and osteoclasts. Chondrocyte growth causes long bones to elongate at both ends and to mineralise at the two endochondral zones (Fig. 1a, right) via a process known as endochondral ossification^9,10^. During this process, osteoclasts support the removal of cartilage matrix^11^ and cooperate with osteoblasts to replace the remaining cartilage with mineralised bone trabeculae^12^. Some osteoblasts are isolated/surrounded by mineralised bone and develop into osteocytes within lacunae (open spaces) to form a connective network of fine canaliculi (mineralised bone channels). The osteocyte network regulates osteoblast and osteoclast differentiation by secreting several factors, including sclerostin and receptor activator of nuclear factor kappa-Β ligand (RANKL)^13^. However, the precise mechanisms underlying the formation of the osteocyte network and canaliculi remain unclear. To study these processes, observations must be made under aqueous conditions because the cells involved, particularly hypertrophic chondrocytes, are usually surrounded by complex matrix chambers filled mostly with aqueous liquid and large amounts of soft collagen-fibres. In the endochondral zone, these are absorbed and replaced by mineralised trabeculae that contribute to the strong, lightweight structure of spongy bone while making space for haematogenesis.

Optical microscopy (OM)^14^ and electron microscopy (EM)^15^ have been used extensively to study bone development; these techniques have enabled great advances. Although OM allows tissues to be observed in aqueous solution, diffraction-limited OM has a resolution limit of 200 nm due to the wavelength of light employed, among other factors. Conversely, although traditional transmission EM (TEM) has a high resolution, the sample must be observed in a vacuum, requiring bone tissue samples for standard EPON-embedding/thin-sectioning TEM to be demineralised and dehydrated using organic solvents before imaging. Environmental scanning EM allows wet samples to be imaged in a low-pressure atmosphere of approximately 1000 Pa (1/100 atm) using differential pumping and gaseous electron detector technologies^16,17^. This technique has enabled samples surrounded by a very thin layer of water to be observed in a low vacuum at temperatures just above 0 °C. To allow wet samples to be imaged in a more stable aqueous environment, environmental capsules have been developed for TEM^18–20^ and scanning EM (SEM)^21^; these capsules have been successfully introduced into vacuum chambers and used to image hydrophilic molecular complexes^22^, cells^23^, and tissues^24,25^. Indeed, Vidavsky et al*.* successfully observed calcium carbonate mineralisation in sea urchin embryos under wet conditions using a B-nano SEM (a field emission SEM column sealed with a SiN film at the bottom end)^26^.

Previously, we developed atmospheric SEM (ASEM)^27,28^ to observe aldehyde-fixed biological samples of various dimensions immersed in aqueous liquid (Fig. 1b). This technology has been used to successfully image neurons that were cultured directly on an open ASEM dish^29–31^ and fixed; in addition, secretory glands^32^, muscles^33^, and bacterial biofilms^34^ have been successfully fixed and imaged using this technology. Moreover, ASEM was recently used to image inorganic calcium phosphate mineralisation in spongy and cortical bone tissues^35^.

Here, we report liquid-phase EM observations of cartilage tissues, including chondrocytes, at different stages of development and endochondral ossification. Tissues immersed in aqueous buffer were imaged by ASEM at high resolution without decalcification or dehydration on the day of or the day after aldehyde fixation. Imaging thick bone sections exposed by a single cut revealed that osteocytes in the endochondral ossification zone were surrounded by variously mineralised extracellular matrices. Furthermore, in mouse femurs lacking the oxidative stress sensor Kelch-like ECH-associated protein 1 (Keap1)^36^, impaired calcification and delayed penetration of blood vessels into the epiphyseal cartilage were revealed by ASEM shortly after fixation.

## Results

### Correlative light-electron microscopy (CLEM) by ASEM with haematoxylin and eosin (HE) staining

**Figure 2:**
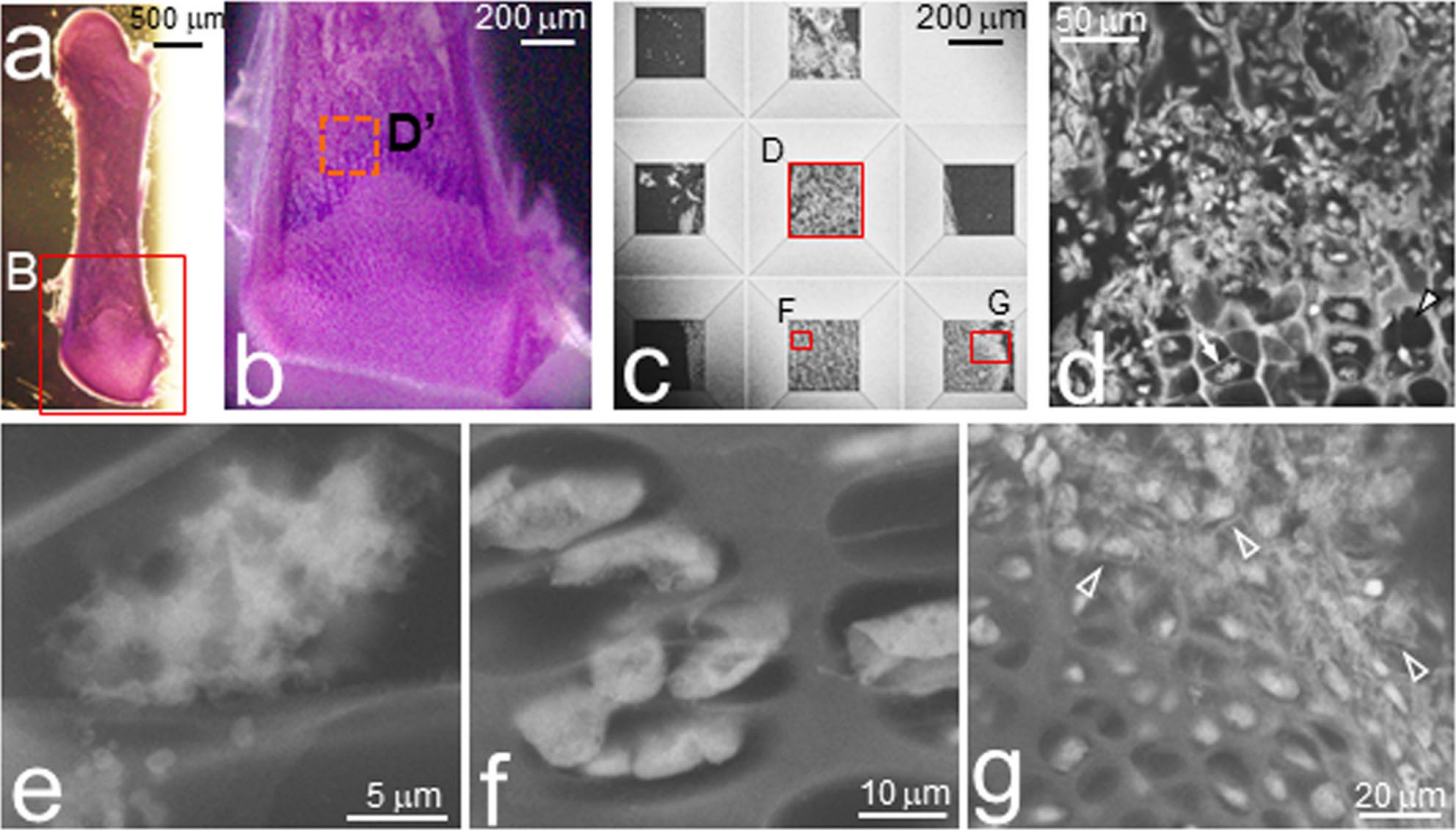
Development of HE-stained correlative light-electron microscopy (CLEM) using ASEM to image tissues. Fixed femurs of P1 mice were directly embedded in 4% agar, sliced into 200-μm-thick sections, and stained with HE. (**a**) Optical microscopy image of a femur section recorded using an optical stereomicroscope. (**b**) High magnification image of the annotated square B in panel (**a**). (**c–g**) ASEM images. (**c**) Low magnification overview image of the femur stained with PTA positioned on the eight SiN film windows^32^ of the ASEM dish. (**d**) High magnification image of window D in panel (**c**) showing the epiphyseal growth plate. (**e**) High magnification image of the area indicated by the arrow in panel (**d**). A hypertrophic chondrocyte with protrusions surrounded by partitions. (**f**) High magnification image of the red square in window F of panel (**c**). Strongly stained proliferating chondrocytes separated by extracellular matrix. (**g**) High magnification image of the red square in window G of panel (**c**). Resting chondrocytes and fibrous structures (open arrowheads) were imaged in the periarticular region.

To determine the precise position of a tissue, particularly bone tissues, we developed a novel CLEM technique using ASEM with HE staining (Fig. 1b,c). Specimens were prepared by aldehyde-fixation of femurs isolated from new-born mice at postnatal day 1 (P1), cut along the longitudinal axis using a vibratome, and stained with HE (Fig. 2a,b). Despite the 200-μm thickness of the specimens, which is unusually thick for tissue OM, different chondrocyte zones were easily identified based on the colour and shape of the cells (Fig. 2b), thus helping to position the target tissue area correctly on the windows of ASEM dishes (Fig. 1c). The tissues were then stained with phosphotungstic acid (PTA), immersed in radical scavenger buffer, and observed by inverted SEM (Figs. 1b and 2c). HE staining did not interfere with PTA staining (Fig. 2c–g) and was always performed.

Figure 2 shows the endochondral zone of a femur section visualised by HE-OM (Fig. 2a–b) and ASEM (Fig. 2c–g). The bone appears bright in the ASEM image of the PTA-stained section (Fig. 2c), whereas the aqueous buffer surrounding the bone appears dark. A comparison of the HE-OM image (Fig. 2b) and the ASEM overview image of the positioned sample (Fig. 2c) revealed that the horizontal central line of window D in the ASEM dish (Fig. 2d and Supplementary Fig. S1) almost corresponded to the junctional zone between the HCZ and the trabecular bone forming zone (TZ), also known as the endochondral ossification growth plate. Lacunae were sometimes prominent and were empty for a depth of 2–3 μm, observable by SEM at an acceleration voltage of 30 kV^29^ (Fig. 2d, white arrowhead). In other compartments, we observed hypertrophic chondrocytes with protrusions and intracellular vacuoles (Fig. 2d, arrow, and Fig. 2e). Figure 2f shows a high magnification image of the region marked in window F (Fig. 2c) containing proliferating chondrocytes with a flat and smooth surface, whereas round resting chondrocytes and convoluted collagen-like fibrous structures (open arrowheads) were observed in a periarticular region (Fig. 2g). These results indicate that HE staining and ASEM successfully imaged the area in which femoral epiphysis growth takes place; therefore, we used the same method to image mouse femurs and tibia at various developmental stages.

### ASEM imaging of femur cartilage and perichondrium at embryonic day (E) 15.5

**Figure 3:**
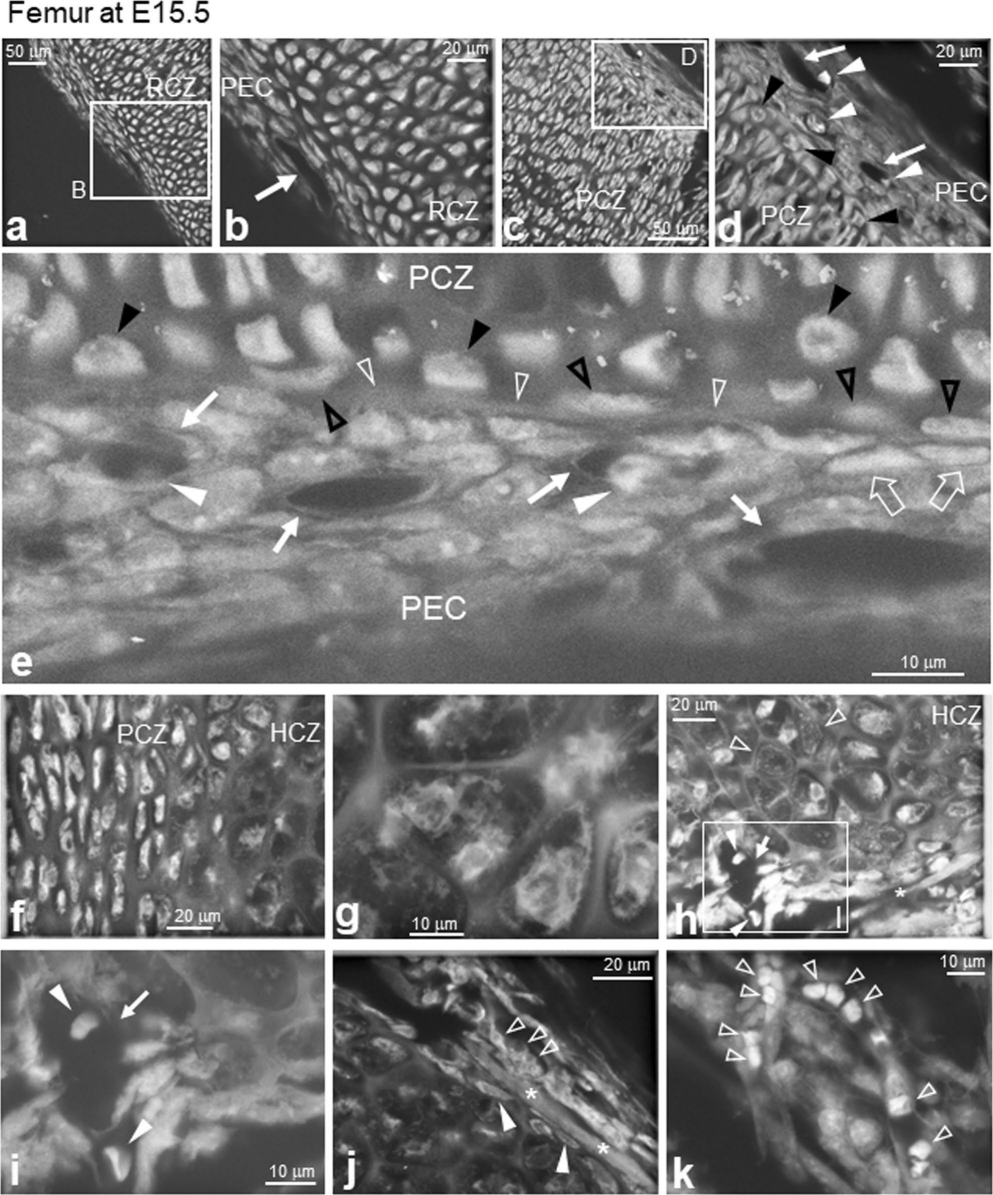
ASEM images of cartilage and primary ossification centre formation in E15.5 femur. Fixed femurs were stained with PTA and imaged by ASEM. (**a**) Low magnification image of a periarticular region. (**b**) High magnification image of the square B in (**a**). Round resting chondrocytes are abundant in the RCZ, which is surrounded by PEC. The PEC is penetrated by blood vessels (arrow). (**c**) Low magnification image of elongated proliferating chondrocytes in the PCZ. (**d**) High magnification image of the square D in (**c**). Blood cells (white arrowheads) can be seen in many blood vessels (white arrows) in the PEC. Unique round cells, each of which had a dark nucleus, were observed on the inner side of the border membrane between the PCZ and PEC (black arrowheads). (**e**) High magnification image of the interface between the PCZ and PEC. Blood vessels had tubular structures formed by vascular endothelial cells (arrows) and contained blood cells (white arrowheads). A novel border layer (white open arrowheads) was present between the PCZ and the PEC. Unique round (black arrowheads) and spindle-shaped (black open arrowheads) cells were directly adjacent to this border structure on the PCZ side. Spindle-shaped cells were observed on the PEC side of the border (white open arrows). (**f**) Low magnification image of the PCZ and the HCZ. Left: elongated proliferating chondrocytes with dark nuclei surrounded by bright cytoplasm. Middle to right: cell compartments started to swell, with fully swollen hypertrophic-chondrocytes in the lacunae (right). (**g**) High magnification image of the HCZ. Swollen cells with sparse contents were surrounded by thick filamentous walls. (**h**) Low magnification image of the interface between the HCZ and the cortical bone collar (*). (**i**) High magnification image of the square I in (**h**). Blood cells (arrowheads), presumably erythrocytes, imaged in the sectioned blood vessel (arrow). (**j**) Continuous collar structure (*) at the edge of the HCZ. Spindle-shaped cells (white arrowheads) existed between the HCZ and bone collar, with mononuclear cells outside of the bone collar (open arrowheads). (**k**) Capillaries containing blood cells (open arrowheads) just outside the bone collar. HCZ, hypertrophic chondrocyte zone; PCZ, proliferating chondrocyte zone; PEC, perichondrium; RCZ, resting chondrocyte zone.

Since skeletal mineralisation starts as early as E15.5^37^, we fixed an E15.5 mouse femur and monitored mineralisation in unstained tissue of distal to proximal regions using ASEM. Although we previously demonstrated that ASEM can image mineralisation as bright signals^35^, we detected no mineralisation at this stage, potentially because of low mineralisation levels at E15.5^37^. When stained with PTA, round resting chondrocytes were imaged in the RCZ of the periarticular region (Fig. 3a) with bright cytoplasm and occasional darker regions attributable to the nucleus (Fig. 3b). Bright signals might reflect very rich cytoplasmic expression since PTA is known to preferentially stain proteins and nucleic acid complexes^27,38^. The RCZ was surrounded by the perichondrium (PEC) and blood vessels (arrow; Fig. 3a,b), whereas flat and laterally elongated cells (i.e. proliferating chondrocytes) were observed at the PCZ when the tissue was scanned towards the proximal side of the bone (Fig. 3c). The proliferating chondrocytes were also surrounded by PEC and blood vessels (Fig. 3d, arrows) in which blood cells were sometimes observed (white arrowheads). Blood vessel endothelial cells (white arrows) and blood cells (white arrowheads) were visualised more clearly at a higher magnification (Fig. 3e).

A previously unreported border layer almost 1-μm-thick (white open arrowheads) was detected between the PCZ and PEC (Fig. 3e), likely due to ASEM allowing the high resolution and in-liquid observation of unshrunk tissues. The interface sheath between the PCZ and the PEC was lined with round cells (Fig. 3d,e, black arrowheads) that displayed a different shape than surrounding proliferating chondrocytes or cells in the PEC. The majority of spindle-shaped cells were located on the PCZ side of the border layer (Fig. 3e, black open arrowheads). Interestingly, some spindle-shaped cells were observed on the PEC side (Fig. 3e, open arrows) near the blood vessel (Fig. 3e, white arrows).

In the neighbouring proximal region, the thick filamentous compartment surrounding each chondrocyte was more swollen and formed a larger internal aqueous space for each cell, identifying this region as the HCZ (Fig. 3f). At a high magnification, variously shaped swollen cells were observed in lacunae (Fig. 3g), and the walls of a blood capillary (arrow) and enclosed blood cells (arrowheads) appeared bright in the HCZ (Fig. 3h,i). The blood cells were flat and round in shape and thus might have been erythrocytes due to their preferential staining by PTA^32^. Faint expanded cells were visible near the blood vessels (Fig. 3h, open arrowheads) and all lacunae appeared to be filled with cells, unlike the empty lacunae observed in other areas of the HCZ (Fig. 2d). A sheath structure with continuous high electron density surrounding the HCZ was identified as a bone collar that later develops into cortical bone (Fig. 3h,j, *). Between the HCZ and the bone collar, we observed spindle-shaped cells (Fig. 3j, white arrowheads) similar to those present at the periphery of the PCZ (Fig. 3e, black open arrowheads) but with a different shape than hypertrophic chondrocytes. A row of mononuclear cells, likely osteoblasts based on their shape, were attached to the outside of the bone collar (Fig. 3j, open white arrowheads), whereas its outer periphery was surrounded by capillaries containing many aligned blood cells (Fig. 3k, open white arrowheads, and Supplementary Fig. S2). Taken together, these findings indicate that ASEM successfully imaged undeveloped vascular invasion, the primary ossification centre, and aqueous volumes.

### ASEM imaging of trabecular- and cortical- bones and cartilage in P1 femur

**Figure 4:**
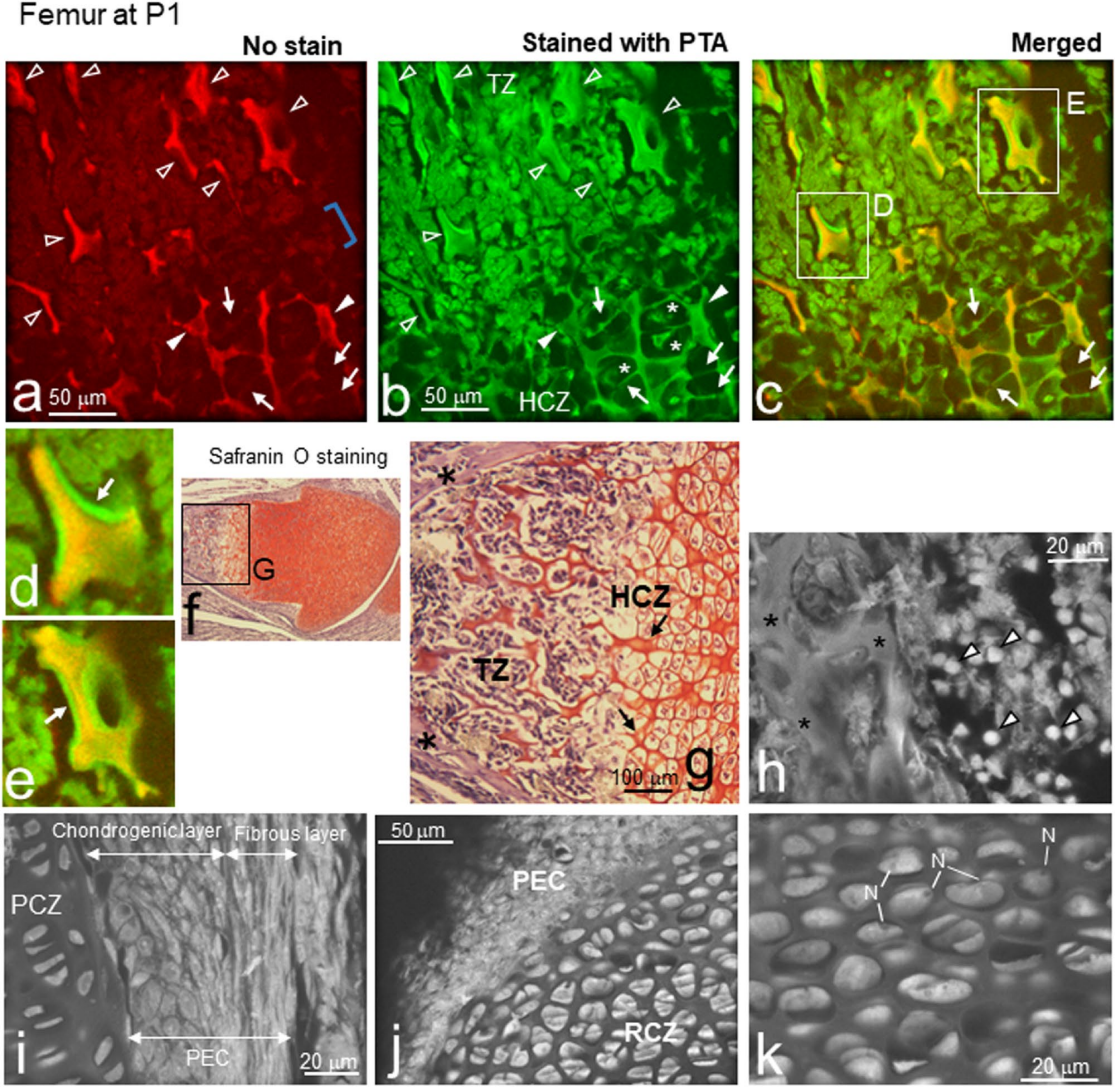
Primary ossification of cartilage forms trabeculae in P1 femurs. The RCZ, PCZ, HCZ, and TZ were imaged using ASEM. (**a**) The transition from the HCZ to the TZ was imaged without PTA staining to detect mineralisation. The bright electron-dense walls imaged in the proximal region are artificially coloured in red using ImageJ version 1.52U (http://rsbweb.nih.gov/ij/) and indicate the presence of CaP-mineralised trabeculae (open arrowheads). The trabecular bone was mineralised throughout its core and had a different appearance than superficially mineralised walls (arrowheads), where a non-mineralised gap (blue square bracket) existed between the TZ and the HCZ. The longitudinal walls were more mineralised than the lateral walls in the HCZ (arrows). (**b**) The same field after PTA staining was artificially coloured in green similarly. The non-mineralised gap was connected by non-mineralised trabeculae and numerous soft cells were clearly visible. (**c**) Merged image of (**a**) and (**b**) created by ImageJ. (**d**) High magnification image of the white square D in (**c**). Highly mineralised trabecular bone core (yellow) and deposited osteoid (arrow) were evident. (**e**) High magnification image of the white square E in (**c**). Another trabecular bone core (yellow) and deposited osteoid (arrow). (**f**) Fixed femurs were decalcified, dehydrated, and embedded in paraffin. Sections 5-μm-thick were stained with haematoxylin and Safranin O. (**g**) High magnification image of square G in (**f**). Safranin O stained proteoglycan red in the HCZ and TZ, but hardly in cortical bone (*). (**h**)**–**(**k**) Tissues were stained with PTA. (**h**) High magnification image of the TZ. Trabeculae (*) were surrounded by numerous cells. The small round cells with high electron density could be haematopoietic cells (arrowheads). (**i**) Image of the PCZ and the chondrogenic and fibrous layers of the PEC. (**j**) Low magnification image of the PEC and RCZ. (**k**) High magnification image of the RCZ. A dark nucleus (N) was surrounded by very bright cytoplasm, suggesting rich protein production in the cytoplasm. HCZ, hypertrophic chondrocyte zone; PEC, perichondrium; PCZ, proliferating chondrocyte zone; RCZ, resting chondrocyte zone; TZ, trabecular bone zone.

During endochondral ossification, the extracellular matrix produced by chondrocytes is calcified and hypertrophic chondrocytes undergo terminal apoptosis. The zone where hypertrophic chondrocytes underwent apoptosis is then invaded by blood vessels (Figs. 1a and 3) that introduce osteoblast progenitors, which generally initiate trabecular bone formation in long bones by P1^39^. We scanned P1 femurs prior to PTA staining to identify highly mineralised areas using ASEM. Very bright walls of high electron-density were observed in a zone more proximal than the HCZ, suggesting that they were the highly mineralised trabeculae of the TZ (Fig. 4a artificially coloured in red, open arrowheads). In the more distal region (Fig. 4a, bottom right), another moderately electron-dense structure was observed comprised of closed compartments (Fig. 4a, white arrowheads). Interestingly, only the outer wall surfaces were relatively electron-dense (bright), whereas the inner cores had a low electron density, likely reflecting the onset of surface mineralisation. Counterstaining the same area with PTA revealed that moderately calcified chambers sometimes included hypertrophic chondrocytes (Fig. 4a,b and the merged c, *). Interestingly, the lateral walls were barely mineralised (Fig. 4c, green walls indicated by arrows compared with a and b; Supplementary Fig. S3a, arrows) compared with the more mineralised longitudinal walls (Fig. 4c yellow walls), resulting in imperfectly mineralised capsules.

In contrast, in the TZ, the bones were surrounded by many cells (Fig. 4b artificially coloured green and Supplementary Fig. S3a) of low electron density (Fig. 4a) indicating non-mineralized cells. Conversely, the very bright yellow cores in the merged image suggest the formation of highly calcified trabecular bone (Fig. 4c). The surrounding substances (Fig. 4d,e, green layer indicated by arrows) seem to be newly deposited osteoid. As the trabeculae formed are discontinuous and irregular, they constitute primary trabecular bone. In another unstained femur, the cortical bone was also somewhat bright reflecting moderate calcification (Supplementary Fig. S4a). Interestingly, trabecula cores were sometimes brighter than their surroundings, that were interpreted as remaining cartilage residues of high calcification, as shown by Jing et al. using SEM^5^.

To compare the above images with typical OM images using paraffin embedding, other P1 femurs were decalcified, dehydrated, paraffin-embedded, thin-sectioned, and stained with HE. Using OM, the heavily calcified cartilage matrix cores were light blue (Supplementary Fig. S4b and c, arrows), while osteoid covering them was pink (Supplementary Fig. S4b and c, arrowheads)^40^, which is consistent with the ASEM images. When other decalcified thin sections were stained with Safranin O, the proteoglycan of epiphyseal and metaphyseal cartilage matrix was imaged red using OM (Fig. 4f,g). The red area representing metaphyseal cartilage, partly corresponds to the mineralised region observed without PTA staining using ASEM (Fig. 4a). The red surface layers of collagen walls in the HCZ (Fig. 4g, arrows) correspond to the bright ASEM images of surface calcified zones (Supplementary Fig. S4a, arrowheads). Two continuous outer layers sandwiching the HCZ and TZ were not stained with Safranin O, indicating cortical bone (Fig. 4g, *). In addition, mineralised walls in the HCZ sometimes enclosed an aqueous space (Fig. 4b), whereas numerous cells of different shapes and sizes were attached to the trabeculae (Fig. 4h, *). A subset of cells that were relatively small and strongly stained with PTA appeared to be haematopoietic cells, suggesting that blood vessels had penetrated this area of the bone (Fig. 4h, white arrowheads). Moreover, some of the cells could have been osteoblasts or osteoclasts that had differentiated from progenitors introduced via blood vessels (Fig. 3).

In the distal region adjoining the HCZ, the PCZ was surrounded by PEC, which was divided into a chondrogenic layer and a fibrous layer (Fig. 4i). In the more distal RCZ of the articular region, there were many round resting chondrocytes, each of which was surrounded by slightly brighter extracellular matrix than that observed at E15.5 (Figs. 4j, 3a,b), suggesting that it was more developed. At higher magnification (Fig. 4k), subcellular structures were observed within the resting chondrocytes, consisting of a dark nucleus surrounded by bright cytoplasm with a higher electron density, presumably reflecting abundant protein and RNA levels. In addition, a thick layer of PEC cells surrounded the RCZ at the articular surface (Fig. 4j). These results indicate that ASEM successfully imaged trabecular and cortical bone mineralisation without PTA staining, as well as surrounding cells and soft tissues with additional PTA staining.

**Figure 5:**
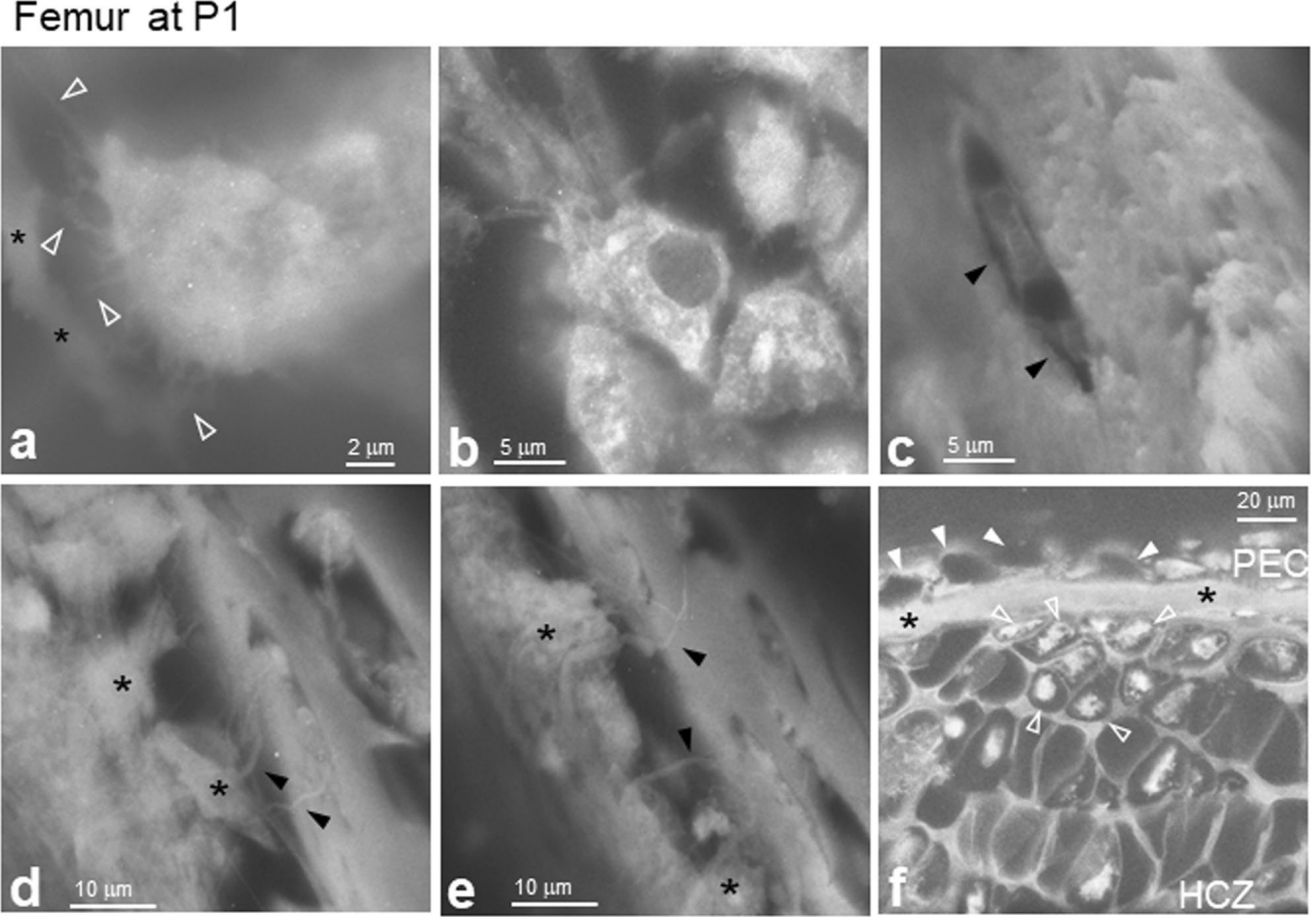
Osteocytes embedded in bone and surrounding cells in P1 femurs. Tissues were stained with PTA and imaged using ASEM. Corresponding low magnification images are shown in Supplementary Fig. S3. (**a**) High magnification (× 8000) image of a cell with protrusions (open arrowheads) towards trabecular bone (*). (**b**) Cells attached to trabecular bone. Various organelles surrounding the nucleus were visible in each cell. (**c**) Osteocytes (arrowheads) in cortical bone. (**d–e**) Other osteocytes in cortical bone. Filamentous structures (arrowheads) connected cortical bone lacuna with external osteoblast-like cells (*). (**f**) Blood vessels (white arrowheads) in the PEC on the outer surface of cortical bone (*) and smaller hypertrophic chondrocytes in the HCZ (open arrowheads). PEC, perichondrium; HCZ, hypertrophic chondrocyte zone.

In the TZ, we successfully imaged large cells with ruffled structures (open white arrowheads) attached to the trabecula (*; Fig. 5a and Fig. S3a–b) and bright mononuclear cells attached to a mineralised wall (Fig. 5b, Supplementary Fig. S3a and c), both of which were attached to hollow spaces in trabecular bone (Supplementary Figs. S5 and S6). In the cortical bone area, cells with protrusions that were assumed to be osteocytes were enclosed and isolated by bone lacunae (Fig. 5c, black arrowheads, Supplementary Fig. S7). The surface of the surrounding bone was rough from sectioning, indicating that the bone had a fibrous internal structure. Pipe-like structures (black arrowheads) were sometimes observed between osteocytes and osteoblast-like cells (*), which formed a row lining the outer side of the cortical bone (Fig. 5d,e) and these might have been extensions of the lacunar-canalicular system of osteocytes. Moreover, blood vessels penetrated the PEC (white arrowheads) on the outer side of the cortical bone (*; Fig. 5f), and relatively small hypertrophic chondrocytes were observed in small lacunae in the HCZ near the blood vessels (Fig. 5f, open white arrowheads).

### ASEM imaging of developed cartilage and trabeculae in P10 tibia

**Figure 6:**
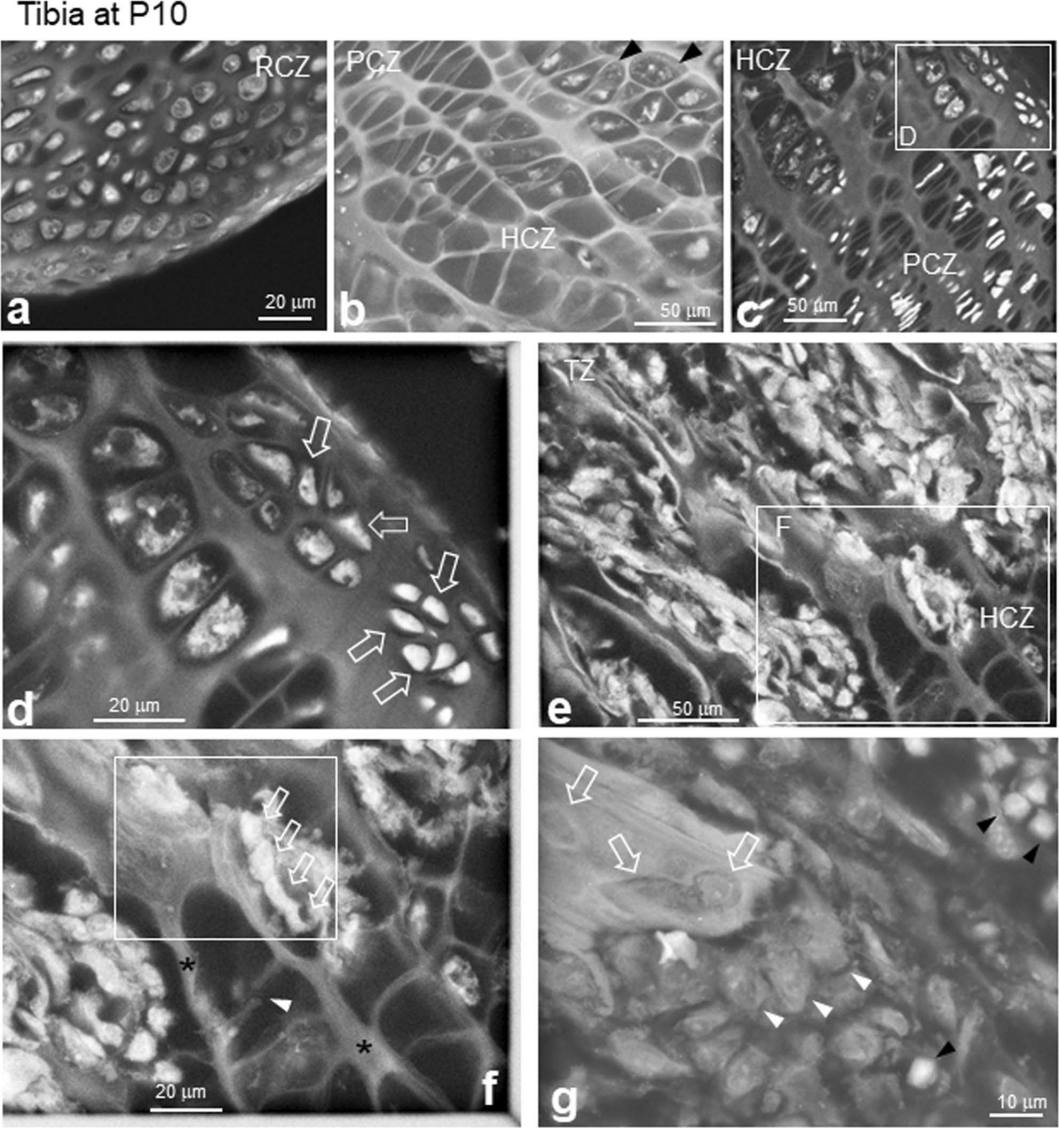
Developed cartilage and trabeculae in P10 tibias. Tissues were stained with PTA. (**a**) RCZ with round resting chondrocytes in the periarticular region showing intracellular structures, including darker nuclei. The cytoplasm of resting chondrocytes was darker (less electron-dense) than that at E15 or P1. (**b**) HCZ. Some empty lacunae were prominent near the endochondral zone. (**c**) Interface between the PCZ and HCZ. (**d**) High magnification image of the white square D in (**c**). Smaller bright cells were present at the bone periphery in the PCZ /HCZ boundary region (open arrows). (**e**) Transition area from the HCZ to the TZ. (**f**) High magnification image of the white square in (**e**). A large number of osteoblast-like cells (arrows) directly faced the calcified longitudinal column (*), with some cracks in the thin lateral connections (white arrowhead). Similar cracks were often observed in such positions as the transition area between the HCZ and the TZ in the P10 femur. (**g**) An area of the TZ. Larger cells with lower electron densities (white arrowheads) and PTA-stained blood cells (black arrowheads). Open arrows indicate resorption pits on the bone surface. RCZ, resting chondrocyte zone; HCZ, hypertrophic chondrocyte zone; PCZ, proliferating chondrocyte zone; TZ, trabecular bone zone.

In the RCZ of PTA-stained P10 tibia, we observed round resting chondrocytes surrounded by a developed extracellular matrix. In each cell, the nucleus was enclosed by moderately dense cytoplasm containing organelles (Fig. 6a and Supplementary Fig. S8a). In the distal side HCZ, some lacunae appeared empty, likely due to apoptosis of hypertrophic chondrocytes^37^ (Fig. 6b). However, expanded hypertrophic chondrocytes with few organelles and low electron density were imaged in proximal region (upper-right) lacuna, which were closer to blood vessels in the PEC (Fig. 6b, black arrowheads). Although proliferating chondrocytes appeared flatter and thinner, the surrounding partition walls formed by the extracellular matrix (Fig. 6c) were thicker than those observed at E15.5 and P1. At the peripheral boundary between the PCZ and HCZ, we observed small, PTA-stained cells with a different shape than proliferating and hypertrophic chondrocytes (Fig. 6c,d, open arrows) with bright cytoplasm surrounding a dark nucleus, similar to the staining pattern observed for proliferating chondrocytes.

In the HCZ and TZ, the total cross section of bone imaged per unit area increased as development proceeded, which might reflect an increase in bone volume (Fig. 6e). Longitudinal columns parallel to the longitudinal bone axis in the HCZ (i.e. trabeculae precursors) appeared to be highly calcified (Fig. 6f, *), whereas the lateral walls were very thin and scarcely mineralised (Fig. 6f, arrowhead). The line of cells observed facing a hollow space in a calcified column were likely osteoblasts (Fig. 6f, white open arrows, and Supplementary Fig. S8b). In another trabecular area, we observed numerous mononuclear cells and larger, less electron-dense cells attached to the bone (Fig. 6g, white arrowheads) and resorption pits (Fig. 6g, arrows), suggesting bone formation by osteoblasts and bone resorption by osteoclasts. Surrounding round cells (Fig. 6g, black arrowheads) might have been haematopoietic cells such as lymphocytes that had been introduced by angiogenesis.

### ASEM imaging of an osteomalacia-like phenotype in Keap1^−/−^ mice at P6

Many signalling proteins, including oxidative stress sensors, are involved in mineralisation. Kelch-like ECH-associated protein 1 (Keap1)^36^, an oxidative stress sensor, is a negative regulator of nuclear factor E2 p45-related factor 2 (Nrf2)^41^, which regulates expressions of various genes including genes of phase 2 antioxidant enzymes. Although *Keap1*^−/−^ newborn mice appeared normal, they unexpectedly died within three weeks. *Keap1*^*−/−*^ mice at postnatal day (P) 7 showed growth retardation; however, there were no histological abnormalities other than hyperkeratosis in the upper digestive tract, which is related to the juvenile lethality^41^. Using *Keap1*^−/−^ P1 mice, we previously demonstrated partial retardation of talar and calcaneus bone formation, shortening of the hypertrophic chondrocyte layer, and a reduced number of osteoclasts *in vivo*^42^.

**Figure 7:**
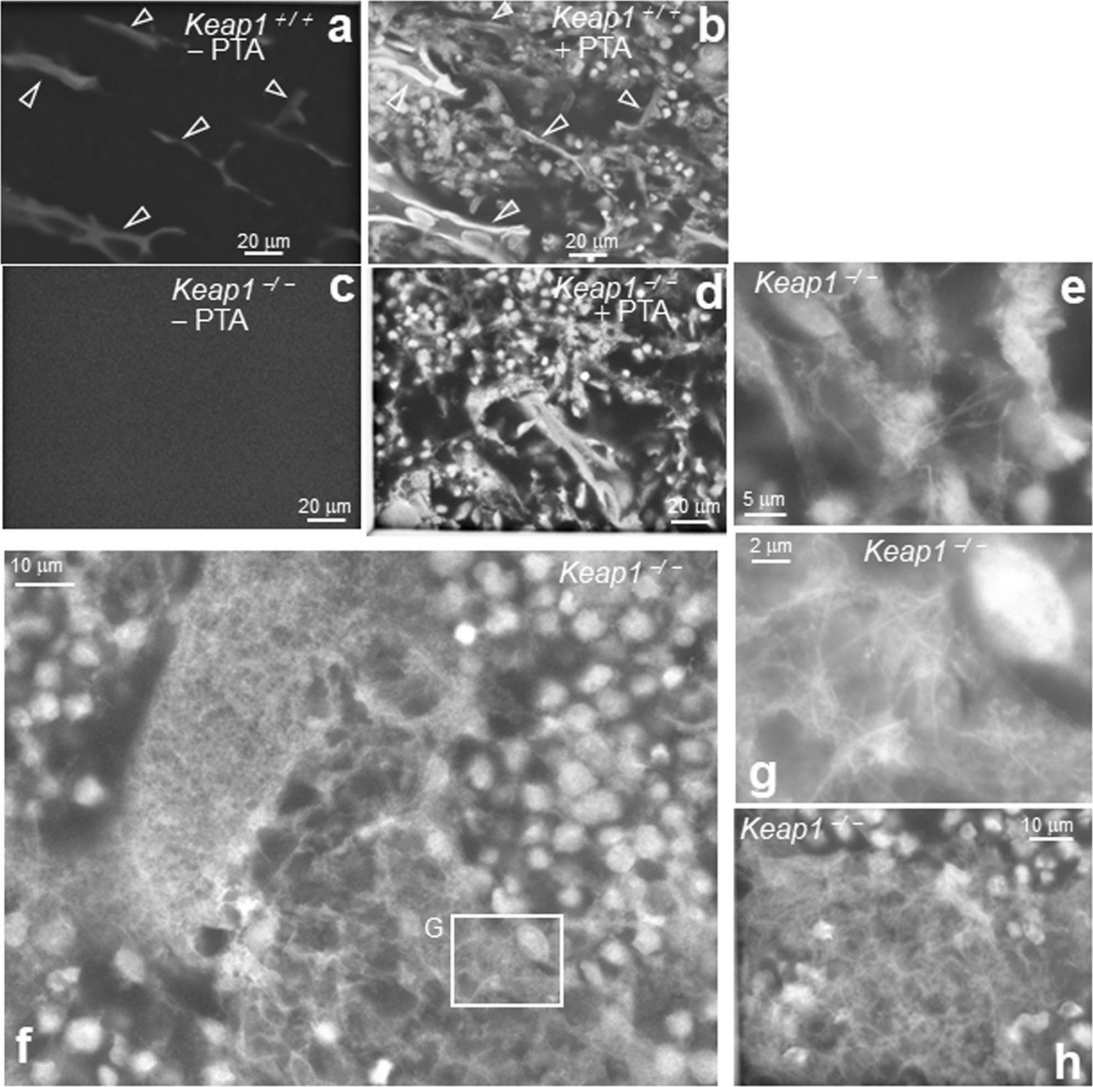
Low mineralisation and abnormal fibrous trabecular bone in P6 *Keap1*^−/−^ femurs. TZ was imaged using ASEM. (**a**) The TZ of a wild-type mouse femur at P6 was imaged without PTA staining. Bright walls of high electron density were observed (open arrowheads) indicating the presence of highly mineralised trabeculae (open arrowheads). (**b**) The same field of (**a**) was stained with PTA. Mineralised trabecular bone (open arrowheads) and numerous surrounding cells were seen. (**c**) The TZ of a *Keap1*^−/−^ femur at P6 was imaged without PTA staining. No bright walls were observed. (**d**) The same field of (**c**) was stained with PTA. Numerous cells and trabecular bone-like structure were present. (**e–h**) Tissues stained with PTA. (**e**) High magnification image of cells and fibrous materials between cells in *Keap1*^−/−^ TZ. (**f**) Abnormal spongy trabecular bone and fibrillary structures in the haematopoietic region of a *Keap1*^−/−^ femur. (**g**) High magnification image of the square G in (**f**). Abnormal sponge-like fibrous structures were present near the trabecular bone in P6 *Keap1*^−/−^ mice femurs. (**h**) Numerous collagen fibrillar structures were present between cells in *Keap1*^−/−^ mice. TZ, trabecular bone zone.

To assess the applicability of the current ASEM technique to hard tissues of genetically modified animals, femurs of *Keap1*^−/−^ mice were examined and compared with the femurs of wild-type (WT) mice at P6. Without PTA staining, very bright walls of high electron density were observed in the TZ of WT femurs at P6 using ASEM as expected (Fig. 7a, open arrowheads and Supplementary Fig. S9a), similar to those in WT femurs at P1 (Fig. 4a). However, no bright signals were observed in the PCZ and RCZ, indicating no calcification (Supplementary Fig. S9b, compared with c). Additional PTA staining revealed numerous cells of various sizes and shapes around the mineralised trabecular bone (Fig. 7b, open arrowheads and Supplementary Fig. S9d–g); some of the attached cells were big, presumably reflecting osteoclasts, while others were small, reflecting osteoblasts.

Conversely, no bright wall was observed in non-stained femurs of *Keap1*^−/−^ mice at P6 (Fig. 7c). PTA staining revealed trabecular bone-like structures attached by several cells of various sizes and with many fibres approximately 200 nm in diameter (Fig. 7d,e, Supplementary Fig. S10). Furthermore, when the observation area was shifted towards a more proximal region, a few spongy abnormal structures without mineral deposits were surrounded by haematopoietic cells and matrices including fibres as thin as 200 nm in diameter (Fig. 7f–h). Thus, high resolution ASEM observation of samples in solution revealed bone hypoplasia and trabecular fibrosis in the femur of the *Keap1*^−/−^ mice, which is an osteomalacia-like phenotype. Immature trabeculae are most likely to be collagen fibres without calcification.

**Figure 8:**
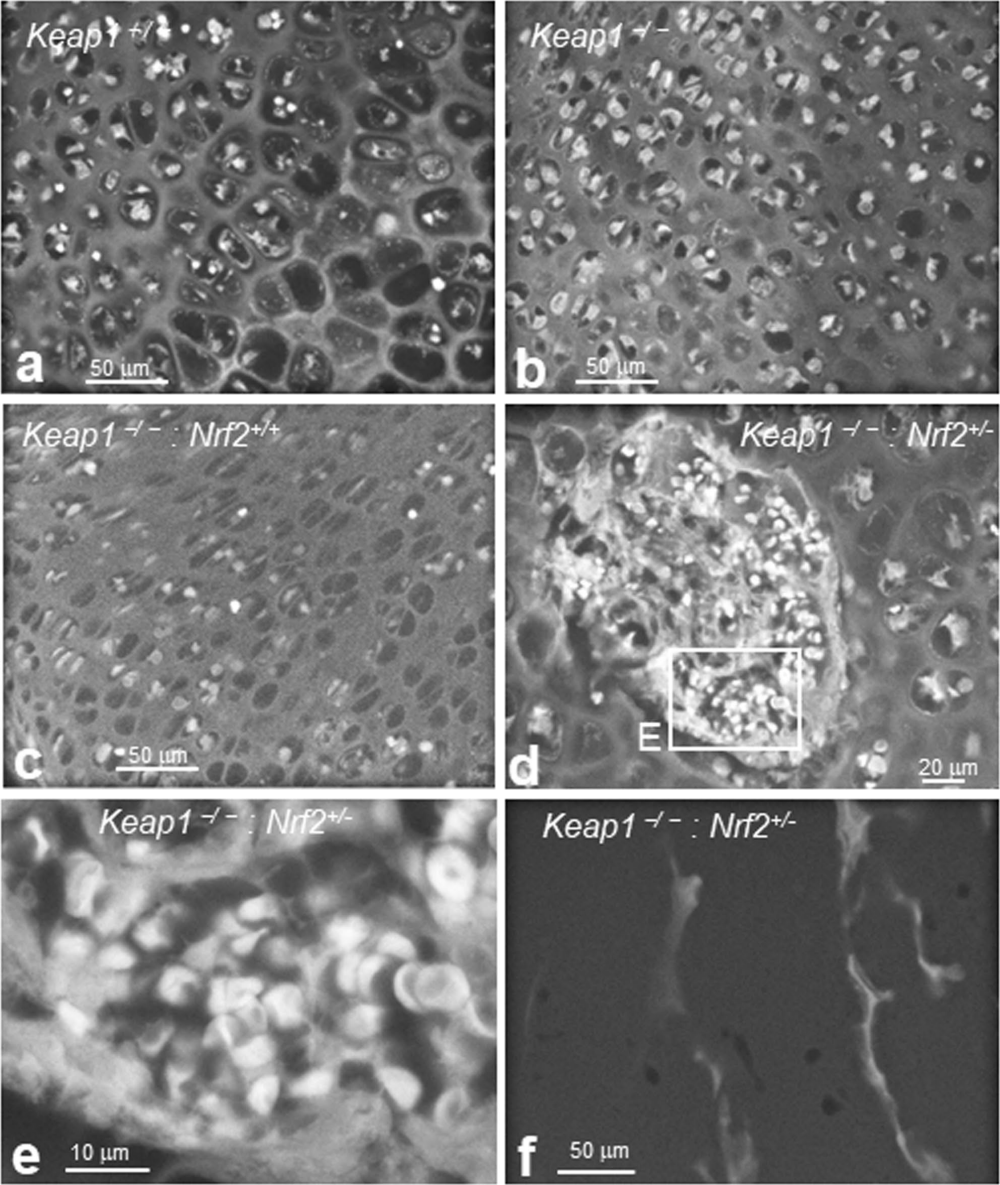
Delayed formation of a secondary ossification centre in P6 *Keap1*^−/−^ femurs is rescued by *Nrf2* heterozygosity. Tissues were stained with PTA and the RCZ was imaged using ASEM. (**a**) Hypertrophied cells in the RCZ of a wild-type femur. (**b**) Hypertrophy of the RCZ was delayed in *Keap1*^−/−^ femurs. (**c**) Vascular penetration of the RCZ was delayed in *Keap1*^−/−^*::Nrf2*^+/+^ femurs. (**d**) Vascular penetration surrounded by hypertrophied cells observed in the RCZ of *Keap1*^−/−^*::Nrf2*^+/−^ mice. (**e**) Higher magnification image of square E in (**d**). Round, biconcave-shaped cells appear to be erythrocytes in penetrated blood vessels, indicating the formation of a secondary ossification centre. (**f**) The TZ of a P6 *Keap1*^−/−^*::Nrf2*^+/−^ femur was imaged without PTA staining. Electron-dense bright walls are clearly visible, indicating mineralised trabeculae. RCZ, resting chondrocyte zone; TZ, trabecular bone zone.

In the epiphyseal RCZ, where the secondary ossification centre forms, resting chondrocytes were hypertrophied in *Keap1*^+*/*+^ femurs at P6 (Figs. 1a, and 8a, right bottom), but not in the corresponding region of *Keap1*^−/−^ femurs (Fig. 8b). In *Keap1*^−/−^*::Nrf2*^+*/*+^ mice, constitutive activation of NRF2 causes juvenile lethality, while *Keap1*^−/−^*::Nrf2*^+*/−*^ mice attain adulthood; therefore, moderate activation of NRF2 occurs in *Keap1*^−/−^*::Nrf2*^+*/−*^ mice. To investigate the role of NRF2 in *Keap1*^−/−^ mice in the formation of the secondary ossification centre, we compared angiogenesis in the RCZ between *Keap1*^−/−^*::Nrf2*^+*/*+^ and *Keap1*^−/−^*::Nrf2*^+*/−*^ littermates at P6. After confirming that resting chondrocytes were not hypertrophied in the femur of *Keap1*^−/−^*::Nrf2*^+*/*+^ mice (Fig. 8c), resting chondrocytes were found to be hypertrophied, and blood vessels had penetrated the centre of the areas showing hypertrophication in the femurs of *Keap1*^−/−^*::Nrf2*^+*/−*^mice as expected (Fig. 8d,e and Supplementary Fig. S11). Moreover, within these vessels, many round, biconcave shaped cells were observed, which could be erythrocytes (Fig. 8d,e and Supplementary Fig. S11a–e). It should be noted that ASEM-imaged unstained femurs of *Keap1*^−/−^*::Nrf2*^+*/−*^ mice had bright trabeculae reflecting mineralisation (Fig. 8f). The blood vessels formation imaged here using ASEM is consistent with that imaged in WT sections using OM at moderate resolution^7,43,44^.

These results indicate that moderate activation of NRF2 in *Keap1* knockout mice rescues the onset of secondary ossification centre formation.

## Discussion

This study is the first liquid-phase EM observation of endochondral ossification using tissue from late embryonic to postnatal stages immersed in a quasi-natural aqueous buffer containing glucose. ASEM allows the water-rich extracellular matrix of the cartilage and bone to be imaged directly without decalcification or dehydration, making this method suitable for the high-resolution observation of bone development with several advantages over traditional methods. For example, ASEM does not require 3 weeks of EDTA decalcification of bone or the following thin sections, which are necessary for typical Epon thin-section TEM and paraffin-thin-section OM. Although traditional SEM does not rely on thin sectioning and can be used to observe the mineralised surface of thick tissue, the sample must be dehydrated and dried before it is exposed to the vacuum of the specimen chamber.

As reported recently^35^ and here, ASEM can directly detect mineralisation without staining, whereas additional PTA staining enables structures surrounding bone mineralisation to be imaged since it generally stains proteins and nucleic acid complexes. The minimum staining protocol takes approximately 10 min and stains the bone surface zone observable by ASEM (the depth of 2–3 μm at an accelerating voltage of 30 kV). In addition, the ASEM images can be recorded immediately after the pretreatment, revealing the intracellular structure of cells and the extracellular matrices. By imaging before and after PTA staining, we revealed the relationship between bone mineralisation and embedded osteocytes, attached osteoblasts and osteoclasts, and extracellular matrices.

Rapid imaging of wet tissue at EM resolution is the most important feature of ASEM. Moreover, ASEM with and without staining clearly distinguished between highly calcified bone cores and the surrounding weakly calcified osteoids in the trabeculae (Fig. 4a–e, Supplementary Fig. S4a-c). This allowed for visualisation of the differences in bone quality, which is comparable to OM images with HE staining (Supplementary Fig. S4b-c). The cores seem to correspond to the highly calcified cartilage residue reported by Jing et al*.*^5^. In addition to physiological bone calcification, pathological ectopic calcium deposits can occur in soft tissues, including blood vessels, heart, and lung, which can damage organs^45,46^. Such calcifications can be efficiently visualised and studied using high-throughput ASEM without PTA staining.

During skeletal development in mice, lacunae in the HCZ at the observable depth tended to be filled with cells at E15.5, whereas many appeared empty at P10, suggesting that the hypertrophic chondrocytes died during this period. Chondrocytes secrete extracellular matrices, such as collagen and proteoglycan, into their surroundings^47^. When chondrocytes become sufficiently hypertrophied and mature, the wall formed by the matrix is calcified and prevents the passage of nutrients to the chondrocytes, causing them to die. After hypertrophic chondrocytes undergo apoptosis^48^, it is generally believed that osteoblasts enter the HCZ and form the bone^6^; however, recent studies have suggested that hypertrophic chondrocytes might survive and become osteoblasts and osteocytes^5,49^. The partial lack of mineralisation of the lateral lacunae walls and the presence of expanded hypertrophic chondrocytes near blood vessels could support this theory of hypertrophic chondrocyte survival. Although studies have reported the existence of unique stem cells and borderline chondrocytes in the growth plate^4,6^, it is still debated whether hypertrophic chondrocytes die, or survive and differentiate into osteoblasts, or whether a minor population of osteoblast progenitors introduced by angiogenesis become osteoblasts.

The quick sample preparation of ASEM benefits the preservation of water-rich structures. It enables observation of tubular blood vessels containing liquid and decreases the risk of changes. In this study, we visualised blood vessels invading the PEC and HCZ at E15.5 and found that cells with a different shape than chondrocytes and perichondrocytes were present at the interface between the cartilage and the PEC region. Although the spindle-shaped cells looked similar to previously reported borderline chondrocytes^6^, it is not yet known whether borderline chondrocytes can also be round. Since high resolution ASEM can distinguish between these cell types, the technology can be used to study these cells and resolve their fate. Indeed, the presence of some spindle-shaped cells near a blood vessel in the PEC region suggests that borderline chondrocytes might be introduced via the vasculature. Borderline chondrocytes localised on the 1-μm-thick border layer are shown in Fig. 3e. After PTA staining, the 1-μm-thick border layer appeared slightly brighter than the surrounding cartilage matrix in the PCZ, which could reflect differences in their components. The role of the 1-μm-thick border layer is not clear, but it may act as a primordium of cortical bone or a scaffold for recruiting borderline chondrocytes from blood vessels. Further investigation is required to determine the role of the border layer.

The small, PTA-stained cells observed at the boundary between the PCZ and the HCZ close to the PEC were similar in appearance to the novel Wnt-responsive cells in the outermost layer of the growth plate^50^. However, the fate of hypertrophic chondrocytes and novel cells, such as borderline chondrocytes and Wnt-responsive cells, remains unclear, as do their possible roles in osteogenesis. High-resolution ASEM imaging could be combined with gold immunolabelling of marker proteins and fluorescent proteins to identify the cells present and to study their behaviour during bone formation.

Osteocytes were found to be embedded in the mouse femur bone matrix at P1 and connected to osteoblasts via thin filaments, consistent with a previous report suggesting that osteocytes might communicate with nearby osteoblasts on the bone surface via direct connections^13^. Recent studies have indicated that osteocytes are involved in numerous processes, such as the regulation of bone remodelling, which influences osteocyte development, and by controlling the activity of osteoclasts and osteoblasts^51^. Osteocytes also act as mechanosensory cells via their intra-bone network^52^ and secrete several factors to osteoblasts to control their osteopontin and osteocalcin secretion, which regulate the memory^53^ and immune system^54^. The relatively high specimen thickness of 2–3 μm^29^ that we were able to observe using ASEM under an acceleration voltage of 30 kV could also be used to observe the significant three-dimensional connections between osteocytes and their surrounding cells.

Involvement of oxidative stress sensors in mineralization has been studied using genetic modifications of Keap1-Nrf2 system in mice. To rescue the juvenile lethality of systemic *Keap1*^−/−^ mice, *Keap1*^−/−^*::Nrf2*^*Flox/Flox*^*::K5-Cre* (NEKO) mice were generated, in which NRF2 expression is eliminated in the oesophagus^55^. NEKO mice exhibited low bone density and a small femur size at 8–10 weeks of age. The results observed in NEKO mice, in NEKO osteoclast primary culture, and in *Keap1*^−/−^osteoblast primary culture suggest that bone hypoplasia in NEKO mice is caused by inhibition of osteoblast differentiation^56^. Furthermore, *Keap1*^+/−^ male mice at 18 weeks of age exhibited significantly increased mineral apposition and bone formation and significantly decreased bone resorption^57^. These results suggest that NRF2 activation induced by Keap1 deficiency affects the bone phenotype; however, a role for Keap1 in endochondral ossification has not been reported. In this study, ASEM observations revealed that *Keap1*^−/−^ femoral trabeculae have scarce mineralisation but fibrous structures at P6. Moreover, no secondary ossification centre is formed at this stage. Obstruction of the oesophagus by hyperkeratosis might cause osteomalacia-like phenotypes, possibly because of inadequate dietary calcium and phosphorus intake, similar to rickets^58^. However, the phenotype is also attributed to the constitutive activation of NRF2 because even surviving adult NEKO mice with double *Keap1–Nrf2* knockout in the oesophagus have low bone density and a small body size^56^. This is the first high-resolution observation of bone development in systemic *Keap1*^−/−^ mice during the endochondral ossification period. ASEM can be used to study different genetically modified mice with varied *Nrf2* expression. As shown here, the mineralised area can be readily observed after fixation using ASEM; therefore, high resolution ASEM with and without staining can be employed for the rapid screening of malcalcified bones of various transgenic or knockout mice, especially for hard tissues of juvenile mice with a lethal genetic modification. ASEM is also expected to be applied to quickly diagnose mineralisation of the ilium.

High-resolution ASEM can also be used to study the texture of bone in mineralisation-related clinical studies. Implant treatment is currently an established option for treating defective prostheses and its success is dependent upon the osseointegration or more advanced osteoconduction of the implant^59^. Both the quantity and quality of connections between the bone surface and implant are important and need to be studied further^60^; however, the mineralisation and texture of the structures formed around implants should be assessed under a loading environment as osseointegration is thought to change dynamically in response to load. Currently, it is extremely difficult to observe bone tissues around implants, yet the high-throughput ASEM method without sample embedding that we developed and demonstrated here could be very useful for such clinical research in vitro. In addition, ASEM could aid in the development of bone-substitute materials by assessing and monitoring their inorganic osseointegration in real-time within Ca^2+^ and PO_4_^3−^ solutions in vitro.

In conclusion, this study demonstrated that the rapid ASEM method could image cartilage tissue and angiogenesis-driven endochondral ossification at high resolution in liquid at atmospheric pressure. Moreover, this study showed that this method could reveal new insights while preserving unshrunk and water-rich structures. ASEM could thus be a powerful tool to study the development of hard tissue.

## Methods

### Animals

C57BL/6 mice were obtained from CLEA Japan (Tokyo, Japan). Breeding pairs of *Keap1*^+/−^ (RBRC01388)^17^ and *Nrf2*^−/−^ mice (RBRC01390)^16^ were provided by the RIKEN BRC through the National Bio-Resource Project of the MEXT, Japan (Tsukuba, Japan). *Keap1*^+/−^ mice were crossed to generate *Keap1* WT and *Keap1* knockout mice. To further investigate the role of *Nrf2* in *Keap1*^−/−^ mice, *Keap1* + */ − *mice were mated with *Nrf2*^−/−^ mice to generate *Keap1*^+/−^*::Nrf2*^+/−^ mice, followed by the generation of *Keap1*^−/−^*::Nrf2*^+/−^ mice. Mice were euthanised at E15.5, P1, P6, or P10 and fixed with 4% paraformaldehyde (PFA; Wako Pure Chemicals, Osaka, Japan) in 0.1 M cacodylate buffer (CB, pH 7.4) at 4 °C for at least 1 day. All animal experimental protocols were approved by the Animal Care and Use Committee of Nagasaki University Graduate School of Biomedical Sciences (Permit 1110170952). All experiments were conducted in accordance with the rules for animal experiments at Nagasaki University.

### Tissue sample preparation for ASEM

Hind limbs were fixed with 4% PFA in 0.1 M CB (pH 7.4) at 4 °C for 1 day and further fixed with 1% glutaraldehyde (GA; Nisshin EM, Tokyo, Japan) in 0.1 M CB at 24 °C for 15 min. The samples were washed several times with double-distilled water (DDW), embedded in 4% agar, and cut with a PRO7 linear slicer (Dosaka, Kyoto, Japan) to obtain 200-μm-thick sections or a single cut tissue. The tissues were stained with aqueous 0.067% haematoxylin solution (Lillie-Mayer’s Haematoxylin, Muto Pure Chemicals, Tokyo, Japan) for 1 min at 24 °C and immediately washed with DDW. The sections were then stained with an aqueous solution of 0.05% eosin (diluted from 1% eosin alcohol solution, Muto Pure Chemicals) for 1 min at 24 °C and washed several times with DDW. Samples were then placed on an SiN-windowed specimen dish and immersed in the observation buffer required for ASEM imaging. An Olympus SZX12 stereomicroscope was used to facilitate these operations. Tissue sections were counterstained with 2% phosphotungstic acid (PTA, TAAB Laboratories Equipment Ltd., Aldermaston, UK) at 24°C^35^ for ASEM.

### ASEM imaging

SEM images were recorded from below the specimen holder using the ClairScope ASEM system (JASM-6200, JEOL, Tokyo, Japan; Fig. 1b). Standard 35-mm ASEM dishes with eight 100-nm-thick (0.25 × 0.25 mm) SiN film windows (Fig. 1b)^33^ were used as specimen holders. Femur or tibia sections were placed on an SiN film window, immersed in radical scavenger observation buffer [10 mg/mL (w/v) d-glucose (Dextrose; MP Biomedicals LLC, Illkirch, France), 1 mM CB (pH 7.4), and 60 mM KCl], and imaged using an optical microscope (Olympus BXFM) positioned above the specimen holder and the inverted SEM of the ASEM at an acceleration voltage of 30 kV (Fig. 1c). The electron dose at the highest magnification was 0.19 e^−^/A^2^, which is less than 0.4% of the dose permitted in low-dose cryo-EM aiming at atomic-resolution single-particle reconstructions.

### Safranin O staining

P1 femurs were fixed in 4% paraformaldehyde in CB at 4 °C for at least 1 day and decalcified in 5% ethylenediaminetetraacetic acid for 3 weeks. The decalcified specimens were dehydrated through an ethanol gradient and embedded in paraffin. Sections of 5 μm length were cut in the longitudinal planes and stained with haematoxylin for nuclei and Safranin O for proteoglycans^5^.

### Artificial colouring

After visualising mineralisation in an unstained tissue slab using ASEM, the slab was stained using PTA, and the same area was imaged again using ASEM. The mineralisation and PTA-stained images were artificially coloured red and green, respectively, using ImageJ, and overlaid to produce the final mixed image.

## Supplementary Information

Supplementary Information

## Data Availability

The data that support the findings of this study are available from the corresponding author upon reasonable request.

## Abbreviations

ASEM: Atmospheric scanning electron microscopy
PTA: Phosphotungstic acid
P: Postnatal day
RCZ: Resting chondrocyte zone
PCZ: Proliferating chondrocyte zone
HCZ: Hypertrophic chondrocyte zone
OM: Optical microscopy
EM: Electron microscopy
TEM: Transmission EM
SEM: Scanning EM
CLEM: Correlative light-electron microscopy
TZ: Trabecular bone forming zone
WT: Wild-type
RANKL: Receptor activator of nuclear factor κB ligand
Keap1: Kelch-like ECH-associated protein 1
Nrf2: Nuclear factor E2 p45-related factor 2
PFA: Paraformaldehyde
GA: Glutaraldehyde
DDW: Double-distilled water

## References

[1] Kronenberg HM (2003). Developmental regulation of the growth plate. Nature.

[2] Olsen BR, Reginato AM, Wang W (2000). Bone development. Annu. Rev. Cell Dev. Biol..

[3] Michigami T (2013). Regulatory mechanisms for the development of growth plate cartilage. Cell Mol. Life Sci..

[4] Mizuhashi K (2018). Resting zone of the growth plate houses a unique class of skeletal stem cells. Nature.

[5] Jing Y (2017). Chondrogenesis and osteogenesis are one continuous developmental and lineage defined biological process. Sci. Rep..

[6] Mizuhashi K, Nagata M, Matsushita Y, Ono W, Ono N (2019). Growth plate borderline chondrocytes behave as transient mesenchymal precursor cells. J. Bone Miner. Res..

[7] Sivaraj KK, Adams RH (2016). Blood vessel formation and function in bone. Development.

[8] Karsenty G (2003). The complexities of skeletal biology. Nature.

[9] Erlebacher A, Filvaroff EH, Gitelman SE, Derynck R (1995). Toward a molecular understanding of skeletal development. Cell.

[10] Long F, Ornitz DM (2013). Development of the endochondral skeleton. Cold Spring Harb. Perspect. Biol..

[11] Karsenty G, Wagner EF (2002). Reaching a genetic and molecular understanding of skeletal development. Dev. Cell.

[12] Mackie EJ, Ahmed YA, Tatarczuch L, Chen KS, Mirams M (2008). Endochondral ossification: how cartilage is converted into bone in the developing skeleton. Int. J. Biochem. Cell Biol..

[13] Bonewald L (2011). The amazing osteocyte. J. Bone Miner. Res..

[14] Nijweide PJ, van der Plas A, Scherft JP (1981). Biochemical and histological studies on various bone cell preparations. Calcif. Tissue Int..

[15] Nakamura H, Ozawa H (1996). Ultrastructural, enzyme-, lectin, and immunohistochemical studies of the erosion zone in rat tibia. J. Bone Miner. Res..

[16] Danilatos GD (1981). The examination of fresh or living plant material in an environmental scanning electron microscope. J. Microsc..

[17] Danilatos GD (1991). Review and outline of environmental SEM at present. J. Microsc..

[18] Abrams IM, McBrain JW (1944). A closed cell for electron microscopy. J. Appl. Phys..

[19] Daulton TL, Little BJ, Lowe K, Jones-Meehan J (2001). In situ environmental cell-transmission electron microscopy study of microbial reduction of chromium(VI) using electron energy loss spectroscopy. Microsc. Microanal..

[20] de Jonge N, Ross FM (2011). Electron microscopy of specimens in liquid. Nat. Nanotechnol..

[21] Thiberge S (2004). Scanning electron microscopy of cells and tissues under fully hydrated conditions. Proc. Natl. Acad. Sci..

[22] Evans JE (2012). Visualizing macromolecular complexes with in situ liquid scanning transmission electron microscopy. Micron.

[23] de Jonge N, Peckys DB, Kremers GJ, Piston DW (2009). Electron microscopy of whole cells in liquid with nanometer resolution. Proc. Natl. Acad. Sci..

[24] Barshack I (2004). Wet SEM: a novel method for rapid diagnosis of brain tumors. Ultrastruct. Pathol..

[25] Kristt D, Nyska A (2007). The wet tissue SEM—a new technology with applications in drug development and safety. J. Toxicol. Pathol..

[26] Vidavsky N (2014). Initial stages of calcium uptake and mineral deposition in sea urchin embryos. Proc. Natl. Acad. Sci..

[27] Nishiyama H (2010). Atmospheric scanning electron microscope observes cells and tissues in open medium through silicon nitride film. J. Struct. Biol..

[28] Nishiyama H (2014). Atmospheric scanning electron microscope system with an open sample chamber: configuration and applications. Ultramicroscopy.

[29] Maruyama Y, Ebihara T, Nishiyama H, Suga M, Sato C (2012). Immuno EM-OM correlative microscopy in solution by atmospheric scanning electron microscopy (ASEM). J. Struct. Biol..

[30] Hirano K (2014). Electron microscopy of primary cell ultures in solution and correlative optical microscopy using ASEM. Ultramicroscopy.

[31] Kinoshita T, Sato C, Fuwa TJ, Nishihara S (2017). Short stop mediates axonal compartmentalization of mucin-type core 1 glycans. Sci. Rep..

[32] Yamazawa T, Nakamura N, Sato M, Sato C (2016). Secretory glands and microvascular systems imaged in aqueous solution by atmospheric scanning electron microscopy (ASEM). Micros. Res. Tech..

[33] Memtily N (2015). Observation of tissues in open aqueous solution by atmospheric scanning electron microscopy: applicability to intraoperative cancer diagnosis. Int. J. Oncol..

[34] Sugimoto S (2016). Imaging of bacterial multicellular behavior in biofilms in liquid by atmospheric scanning electron microscopy. Sci. Rep..

[35] Sato C (2019). Calcium phosphate mineralization in bone tissues directly observed in aqueous liquid by atmospheric SEM (ASEM) without staining: microfluidics crystallization chamber and immune-EM. Sci. Rep..

[36] Itoh K (1997). An Nrf2/small Maf heterodimer mediates the induction of phase II detoxifying enzyme genes through antioxidant response elements. Biochem. Biophys. Res. Commun..

[37] Rafipay A, Berg ALR, Erskine L, Vargesson N (2018). Expression analysis of limb element markers during mouse embryonic development. Dev. Dyn..

[38] Sato C (2019). Primary cultured neuronal networks and type 2 diabetes model mouse fatty liver tissues in aqueous liquid observed by atmospheric SEM (ASEM): staining preferences of metal solutions. Micron.

[39] Tsang KY, Chan D, Cheah SE (2015). Fate of growth plate hypertrophic chondrocytes: death or lineage extension. Develop. Growth Differ..

[40] Ham, A. W. & Cormack, D. H. The development, growth in length and width, and remodelling of long bones in *Histology Chapter 15* (ed. Ham, A. W.) 425–426 (J. B. Lippincott Company, 1979).

[41] Wakabayashi N (2003). Keap1-null mutation leads to postnatal lethality due to constitutive Nrf2 activation. Nat. Genet..

[42] Sakai E (2017). Effects of deficiency of Kelch-like ECH-associated protein 1 on skeletal organization: a mechanism for diminished nuclear factor of activated T cells cytoplasmic 1 during osteoclastogenesis. FASEB J..

[43] Pfander D (2004). Deletion of Vhlh in chondrocytes reduces cell proliferation and increases matrix deposition during growth plate development. Development.

[44] Ma Y (2016). Inactivation of Fam20B in joint cartilage leads to chondrosarcoma and postnatal ossification defects. Sci. Rep..

[45] Murshed M, McKee MD (2010). Molecular determinants of extracellular matrix mineralization in bone and blood vessels. Curr. Opin. Nephrol. Hypertens..

[46] Chan ED, Morales DV, Welsh CH, McDermotto MT, Scwarz MI (2002). Calcium deposition with or without bone formation in the lung. Am. J. Respir. Crit. Care Med..

[47] Noonan KJ, Hunziker EB, Nessler J, Buckwalter JA (1998). Changes in cell, matrix compartment, and fibrillary collagen volumes between growth-plate zones. J. Orthop. Res..

[48] Amling M (1997). Bcl-2 lies downstream of parathyroid hormone-related peptide in a signaling pathway that regulates chondrocyte maturation during skeletal development. J. Cell Biol..

[49] Yang L, Tsang KY, Tang HC, Chan D, Cheah KSE (2014). Hypertrophic chondrocytes can become osteoblasts and osteocytes in endochondral bone formation. Proc. Natl. Acad. Sci..

[50] Usami Y (2019). Possible contribution of Wnt-responsive chondroprogenitors to the postnatal murine growth plate. J. Bone Miner. Res..

[51] Bellido T (2014). Osteocyte-driven bone remodeling. Calcif. Tissue Int..

[52] Turner CH, Forwood MR, Otter MW (1994). Mechanotransduction in bone: do bone cells act as sensors of fluid flow?. FASEB J..

[53] Oury F (2013). Maternal and offspring pools of osteocalcin influence brain development and functions. Cell.

[54] Walsh MC, Takegahara N, Kim H, Choi Y (2018). Updating osteoimmunology: regulation of bone cells by innate and adaptive immunity. Nat. Rev. Rheumatol..

[55] Suzuki T (2017). Hyperactivation of Nrf2 in early tubular development induces nephrogenic diabetes insipidus. Nat. Commun..

[56] Yoshida E (2018). Hyperactivation of Nrf2 leads to hypoplasia of bone in vivo. Genes Cells.

[57] Yin Y, Corry KA, Loughran JP, Li J (2020). Moderate Nrf2 activation by genetic disruption of Keap1 has sex-specific effects on bone mass in mice. Sci. Rep..

[58] Bouillon R, Antonio L (2020). Nutritional rickets: historic overview and plan for worldwide eradiation. J. Steroid Biochem. Mol. Biol..

[59] Li J (2017). Relationships among bone quality, implant osseointegration, and Wnt signaling. J. Dent. Res..

[60] Licata A (2009). Bone density vs bone quality: what’s a clinician to do?. Cleve. Clin. J. Med..

