# Supplementary material for: Liquid-phase ASEM imaging of cellular and structural details in cartilage and bone formed during endochondral ossification: Keap1-deficient osteomalacia
Source: Sci Rep. 2021 Mar 11;11:5722. doi: 10.1038/s41598-021-84202-z (PMC7952587; doi:10.1038/s41598-021-84202-z)
Supplement: Supplementary file 1 — Supplementary Information [file 41598_2021_84202_MOESM1_ESM.pdf]

**Supplementary information**

# Liquid-phase ASEM imaging of cellular and structural details in cartilage and bone formed during endochondral ossification: Keap1-deficient osteomalacia

**Eiko Sakai[1, *], Mari Sato[2], Nassirhadjy Memtily[2, 3], Takayuki Tsukuba[1], and Chikara Sato[2]**

[1]Division of Dental Pharmacology, Department of Developmental and Reconstructive Medicine, Nagasaki University Graduate School of Biomedical Sciences, 1-7-1 Sakamoto, Nagasaki 852-8588, Japan

[2]Health and Medical Research Institute, National Institute of Advanced Industrial Science and Technology (AIST), Central 6, Higashi 1-1-1, Tsukuba, Ibaraki 305-8566, Japan.

[3]Traditional Uyghur Medicine Institute of Xinjiang Medical University, 393 Xinyi Rd, Urumqi 830011, Xinjiang Uyghur Autonomous Region, China.

***Address correspondence to:** Eiko Sakai, Division of Dental Pharmacology, Department of Developmental and Reconstructive Medicine, Nagasaki University Graduate School of Biomedical Sciences, 1-7-1 Sakamoto, Nagasaki 852-8588, Japan, Tel.: +81-95-819-7654; Fax: +81-95-819-7655; E-mail address: eiko-s@nagasaki-u.ac.jp.

**Sakai et al. Supplementary Fig. S1**

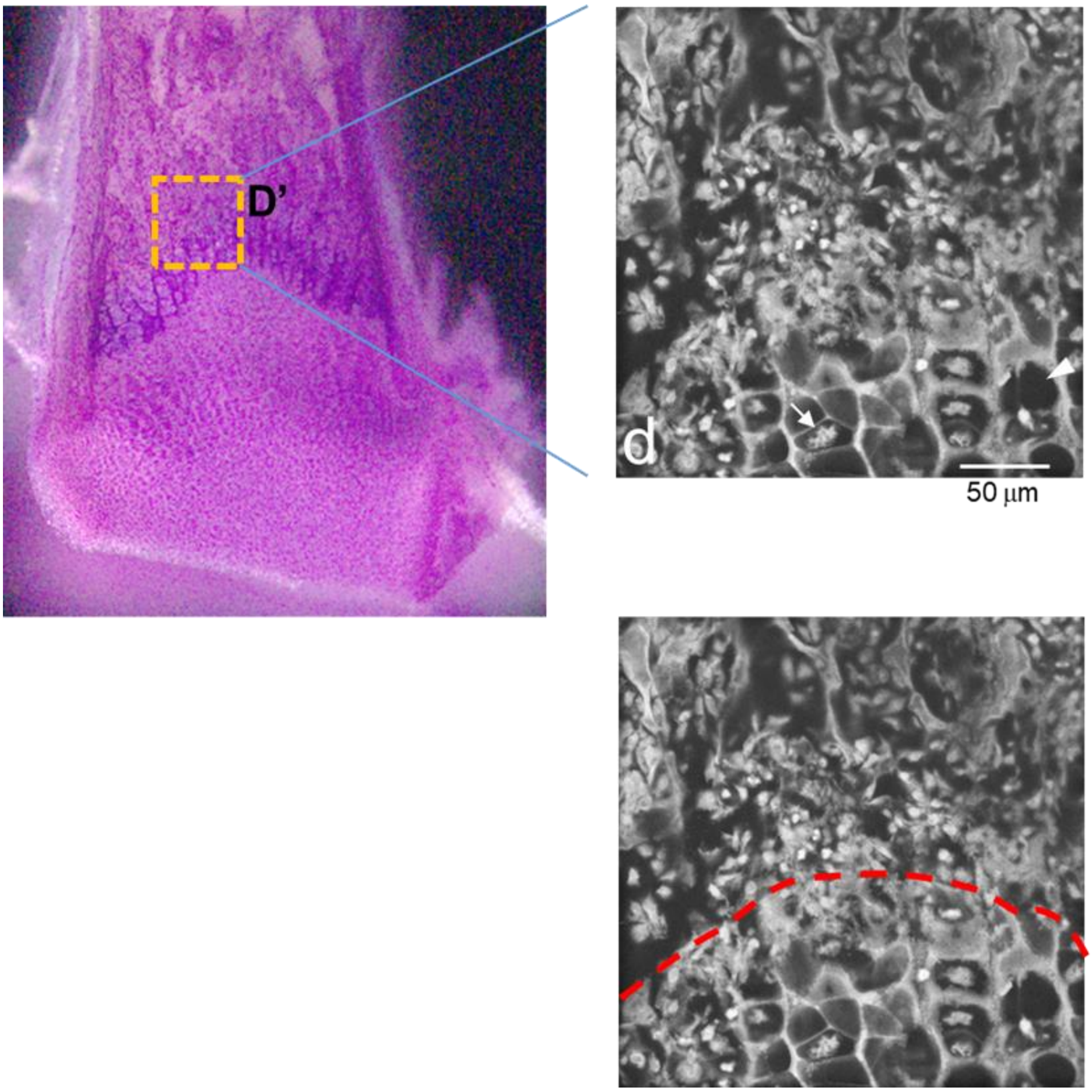


**Supplementary Fig. S1. HE-stained CLEM of P1 femur endochondral zone using ASEM indicating the relationship between Fig. 2b and Fig. 2c–d.** The area shown in the central orange window in the HE-OM image from Fig. 2b (left) corresponds to the high-magnification SEM image of the PTA-stained tissue (upper right, Fig. 2d). The horizontal central red line in the identical SEM image (lower right), indicates the junctional zone between the HCZ and TZ, also known as the endochondral ossification growth plate (EOGP). In the HE-stained OM image, nuclei and trabeculae were stained purple. In the PTA-stained ASEM image, bone appears bright and the aqueous buffer outside the bone appears dark.

**Supplementary Fig. S2**

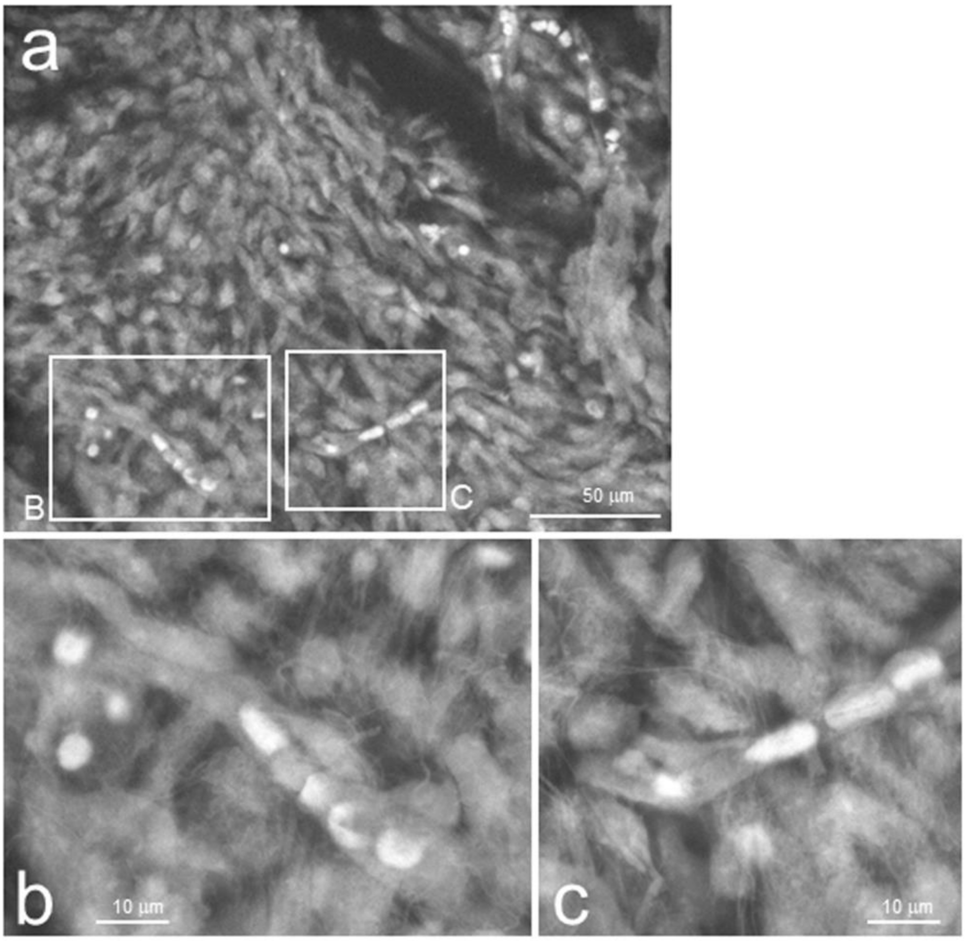


**Supplementary Fig. S2. Capillaries including aligned blood cells outside the bone collar of E15.5 femurs.** Fixed femurs were sectioned, and stained with PTA. (**a**) Low magnification image including Figure 3k. Capillaries including bright erythrocytes are evident. (**b**) High magnification image of the square B in **a**. (**c**) High magnification image of the square C in **a**. Several blood vessels penetrated the periosteum around the bone collar.

**Supplementary Fig. S3**

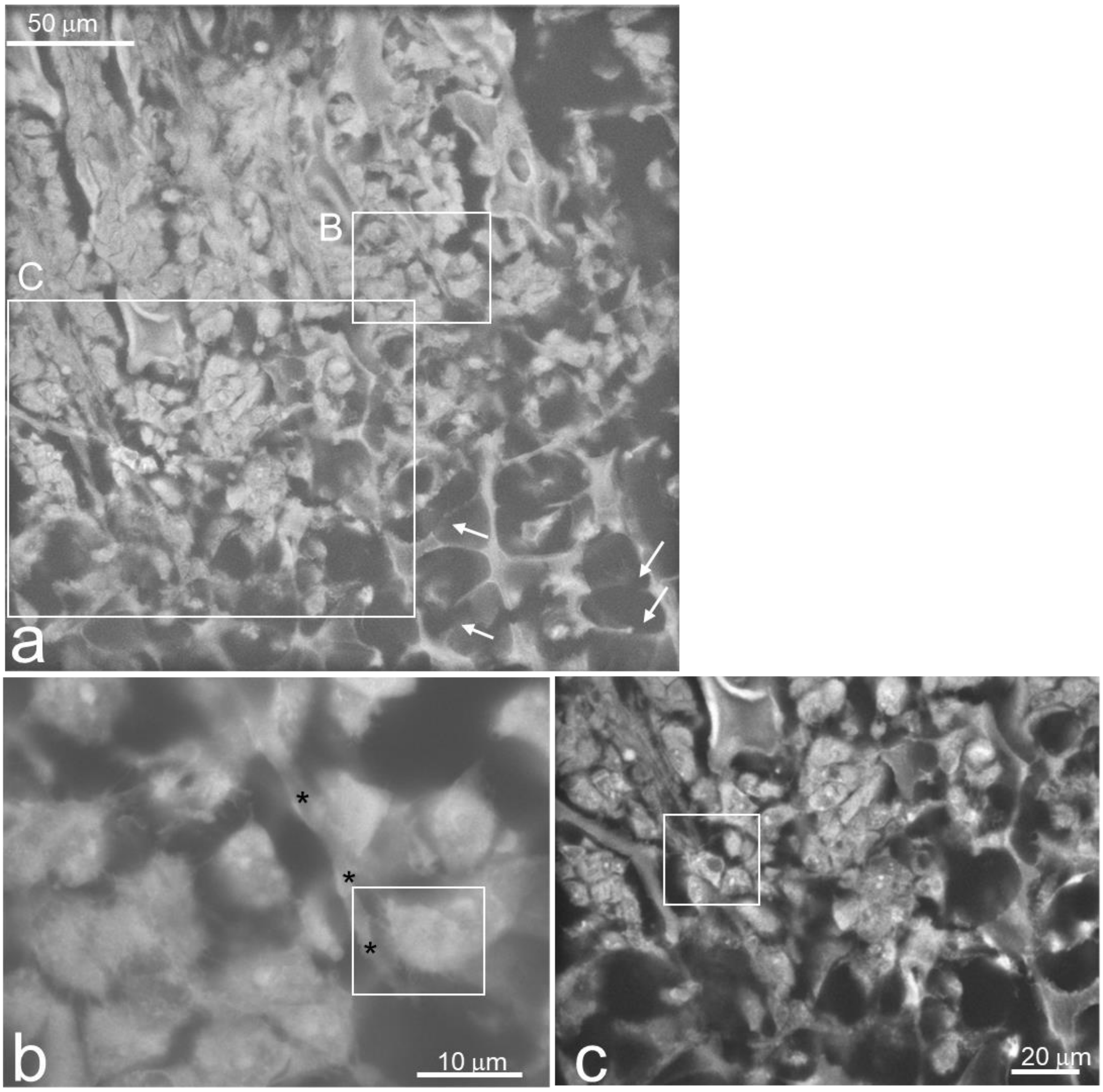


**Supplementary Fig. S3. Cartilage and trabecular bone with surrounding cells in P1 femurs.** Fixed tissue slabs were stained with PTA. (**a**) The original image shown in Fig. 4b but without pseudo-colour. Areas that were imaged at a higher magnification marked by squares. Arrows indicate the lateral walls with lower mineralisation. (**b**) High magnification image of the white square B in **a**. Cells are attached to the trabecular bone (*) via their protrusions. A high magnification image of the white rectangle in **b** is shown in Fig. 5a. (**c**) High magnification image of the white rectangle C in **a**. A higher magnification image of the white square in **c** is shown in Fig. 5b.

**Supplementary Fig. S4**

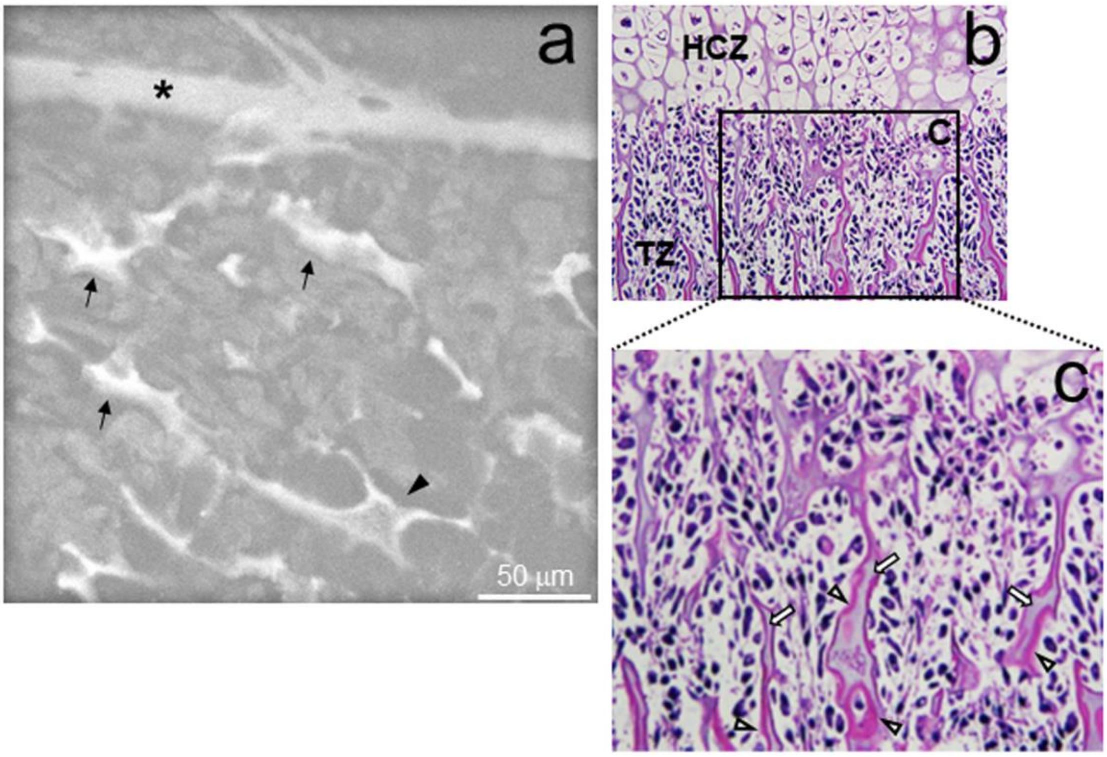


**Supplementary Fig. S4. Calcified cartilage matrix of trabecular and cortical bones of P1 femurs.** Unstained ASEM (**a**) and HE-stained OM (**b, c**). (**a**) ASEM image. Independently prepared mouse femurs were fixed, and sectioned into 200-µm-thick slabs. The section surface was directly imaged without PTA staining using ASEM. The surrounding trabecular bone sometimes appeared bright (arrowhead). Besides, the trabecular bone cores, which correspond to the light-blue trabecular cores stained with HE in b, were sometimes brighter (arrows) than surrounding osteoid and cortical bone (*), suggesting that the cores are cartilage residues[1]. (**b**) OM image of HE-stained tissue thin-section. Mouse femurs were fixed in 4% paraformaldehyde and decalcified in 10% ethylenediaminetetraacetic acid for 3 weeks. The tissues were then dehydrated through an ethanol gradient and embedded in paraffin. Specimens were cut into 5-µm-thick slices parallel to the longitudinal axis of the bone and stained with HE. Images of HCZ and TZ were obtained using a reverse-phase microscope (Olympus, Tokyo, Japan). (**c**) High magnification image of square C in **b**. HE staining coloured the calcified cartilage cores light blue (arrows), while the surrounding osteoid was pink (arrowheads).

**Supplementary Fig. S5**

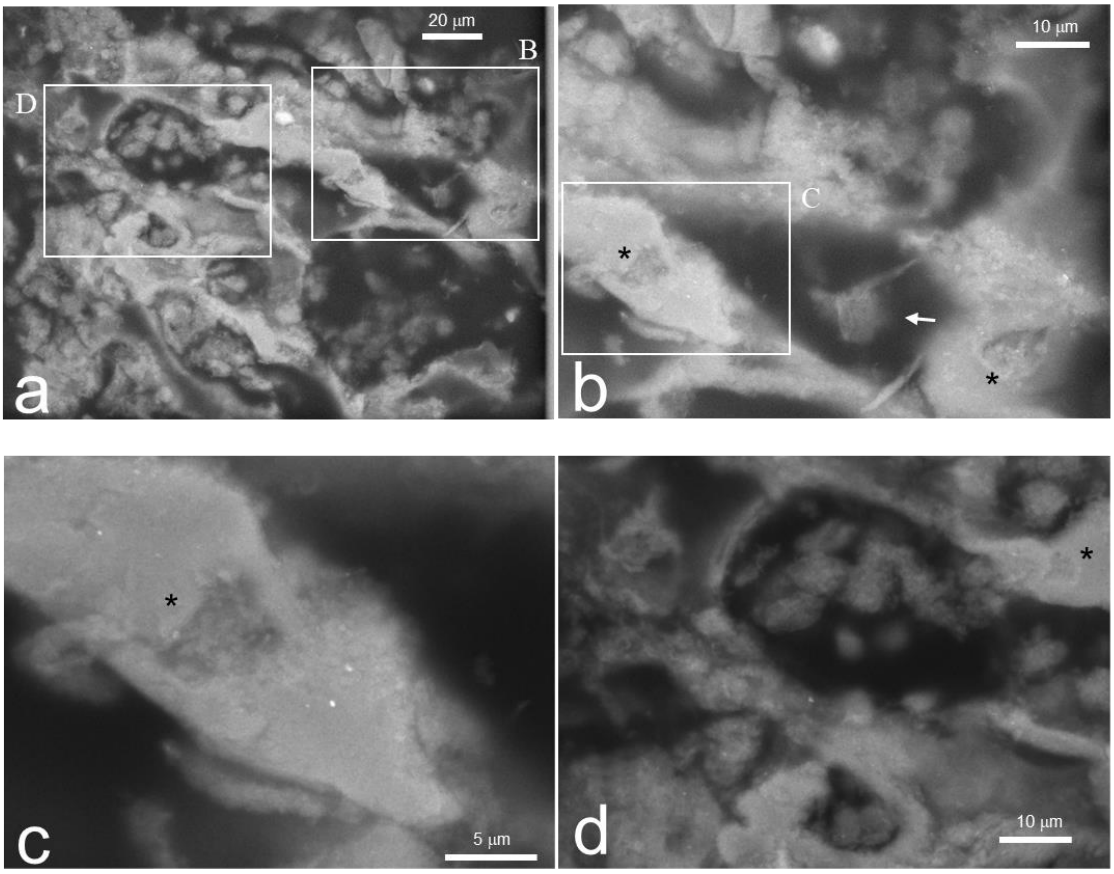


**Supplementary Fig. S5. Spongy bone in the epiphysis growth plate of P1 tibias.** Fixed tissue slabs were stained with PTA. (**a**) Mineralised spongy trabecular bone and surrounding cells, with areas that were imaged at a higher magnification marked as squares. (**b**) High magnification image of white rectangle B in **a**. A cell with low electron density adheres to bone via a fibrous structure (arrow). Hollows on the trabecular bone could be resorption pits formed by osteoclasts (*). (**c**) High magnification image of white rectangle C in **b**. Resorption pit (*). (**d**) High magnification image of white rectangle D in **a**. Trabecular bone with a resorption pit (*) is surrounded by variously shaped cells.

**Supplementary Fig. S6**

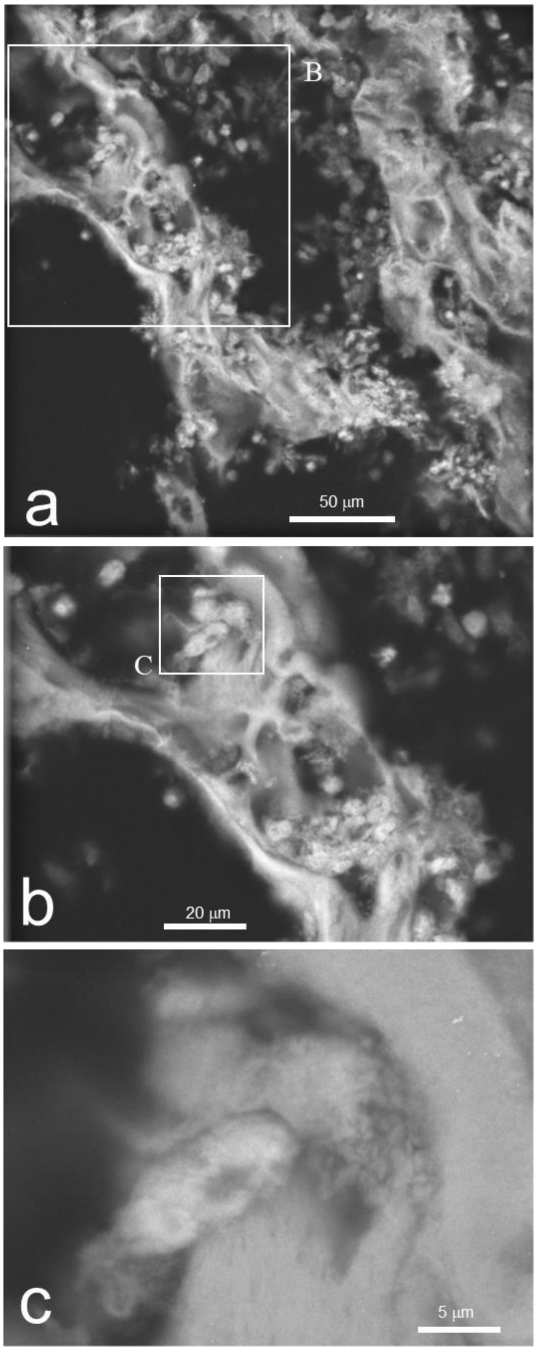


**Supplementary Fig. S6. Spongy bone in another area of P1 tibias.** Fixed tissue slabs were stained with PTA. (**a**) Trabecular bone and surrounding cells. (**b**) High magnification image of white square B in **a**. Trabeculae were attached via many large cells. (**c**) High magnification image of white square C in **b** showing a multinuclear cell and trabecula with extracellular vesicle-like structures in between, along with a neighbouring cell with multiple black nuclei. These cells might be osteoclasts.

**Supplementary Fig. S7**

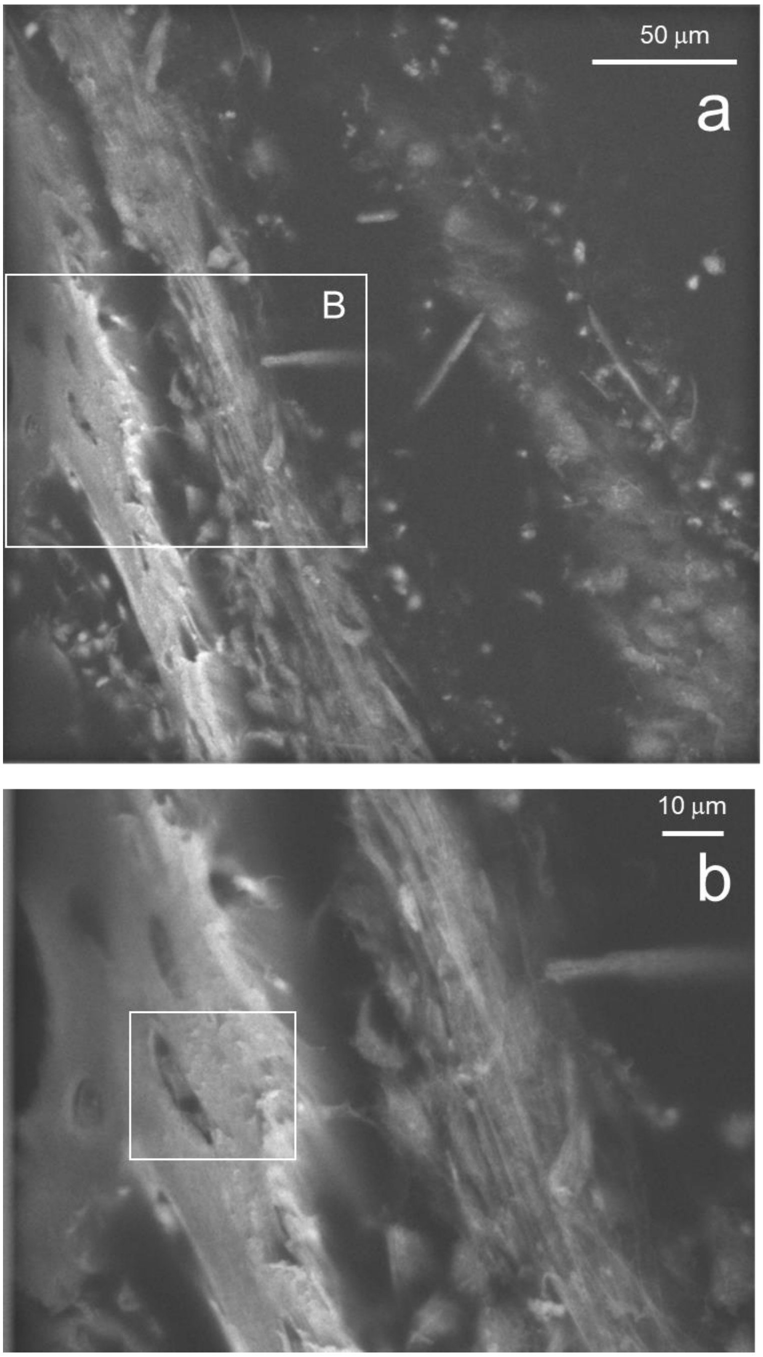


**Supplementary Fig. S7. Cortical bone and the surrounding PEC of P1 femurs.** Fixed tissue slabs were stained with PTA. (**a**) Continuous calcified cortical bone structure with high electron density and osteocytes in its lacunae. (**b**) High magnification image of white square B in **a**. A high magnification image of the white square in **b** is shown in Fig. 5c.

**Supplemental Fig. S8**

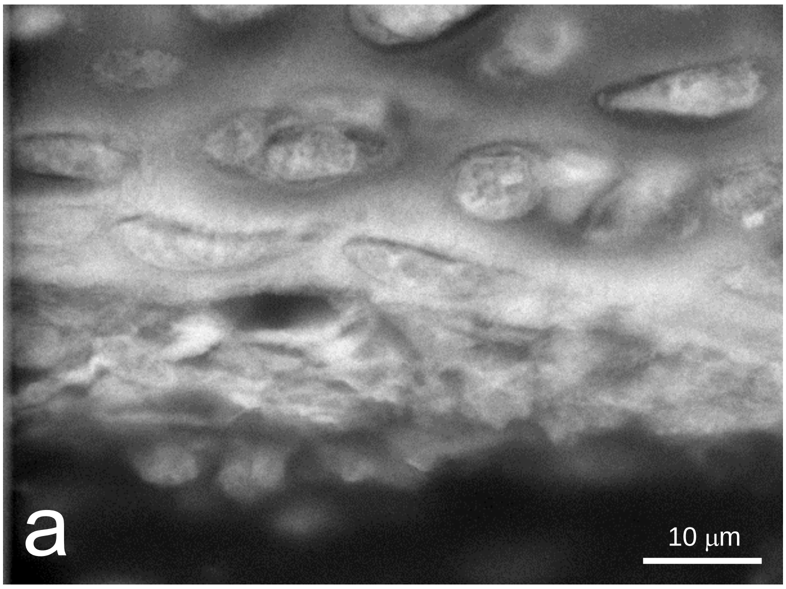


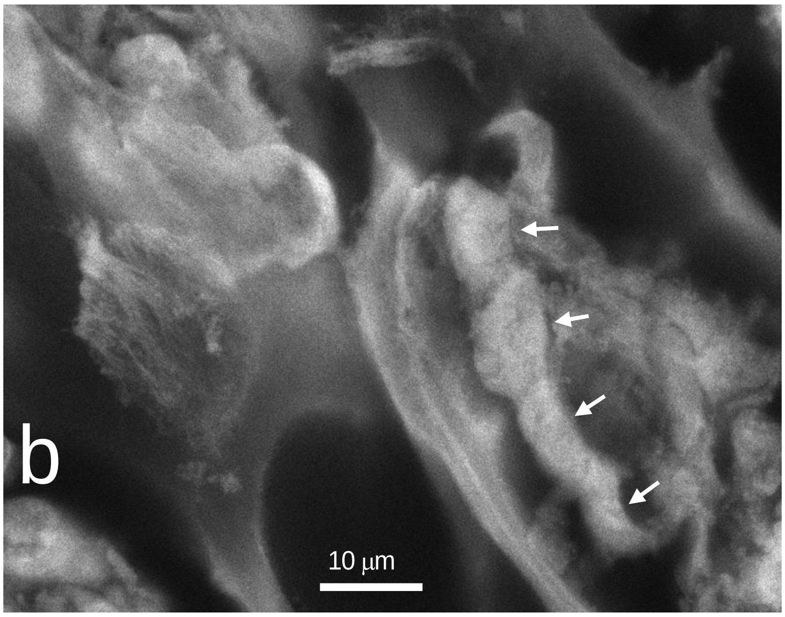


**Supplementary Fig. S8. Resting chondrocytes and osteoblast-like cells in P10 tibias.** Fixed tissue slabs were stained with PTA. (**a**) High magnification image of resting chondrocytes with a large number of cellular organelles. (**b**) High magnification image of the square in Fig. 6f, showing a trabecula attached via flattened large transparent cells (arrows), which might be osteoblasts.

**Supplemental Fig. S9**

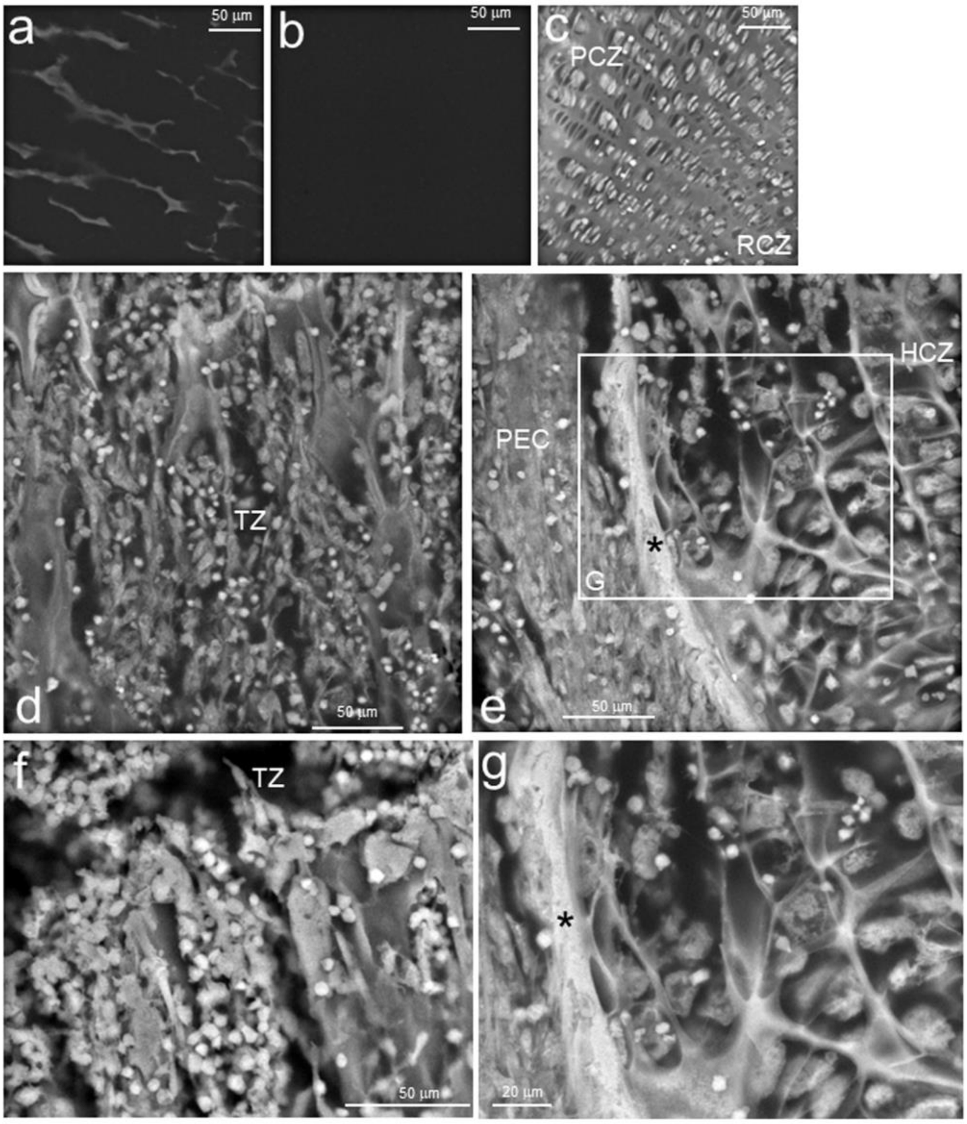


**Supplementary Fig. S9. Highly mineralised trabecular zone in P6 wild-type femurs.** Fixed tissue slabs were imaged using ASEM. (**a**) Low magnification image, including Figure 7a. Electron-dense walls were clearly visible in TZ without PTA staining. (**b**) An area distal of **a** without PTA staining. In the distal direction, no bright walls were observed. (**c**) The same area as **b** after PTA staining. The transition from the RCZ to the PCZ was clearly imaged. No clear signal was detected in these zones by ASEM before PTA staining, indicating non-calcified tissue. (**d-g**) Tissues after PTA staining. (**d**) TZ. Numerous cells attached to trabeculae were imaged. (**e**) Area between the HCZ and PEC. Cortical bone with high electron density (*) was imaged. (**f**) TZ. Bright and round-shaped blood cells and numerous cells of different shapes were attached to the

trabecular bones. (**g**) High magnification image of white square G in **e**. A small cell embedded in cortical bone (*), and hypertrophied cells were imaged in the cartilage lacunae.

**Supplemental Fig. S10**

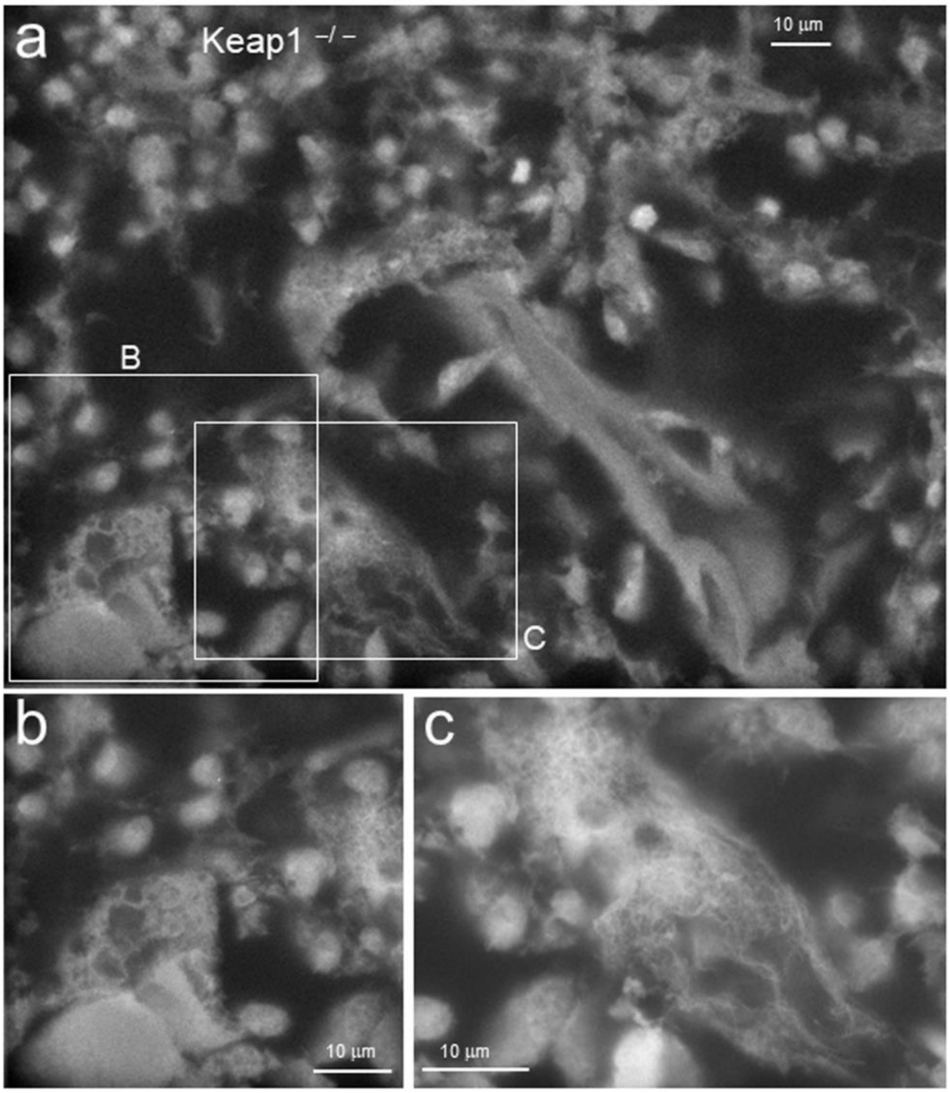


**Supplementary Fig. S10 Fibrous structures in trabecular zone of P6 *Keap1*$^{-/-}$ femurs shown in Figure 7d.** Fixed tissue slabs of *Keap1*$^{-/-}$ mouse femurs at P6 were stained with PTA as in Figure 7d, and imaged using ASEM. (**a**) Higher magnification image of a part of Figure 7d. (**b-c**) Higher magnification images of the annotated squares in **a**. Abnormal fibrous structures were present near the trabecular bone.

**Supplemental Fig. S11**

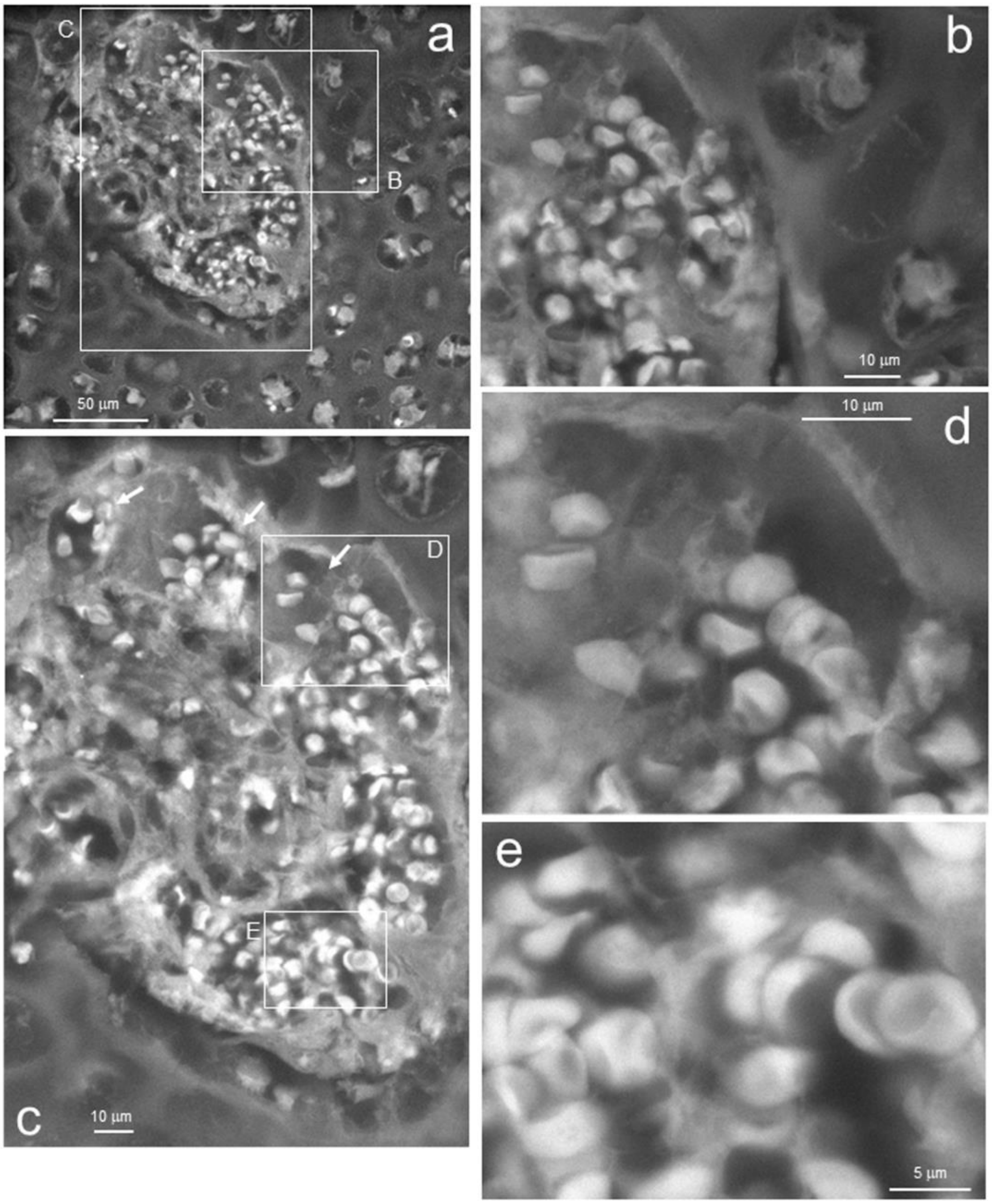


**Supplemental Fig. S11. Angiogenesis in the resting chondrocyte zone of P6 *Keap1$^{-/-}$::Nrf2$^{+/-}$* femurs.** Fixed tissue slabs of *Keap1$^{-/-}$::Nrf2$^{+/-}$* mouse femurs at P6 were stained with PTA, and the angiogenesis areas in the resting chondrocyte zone were imaged using ASEM. (**a**) Low magnification image of Figure 8d. An assembly of multiple blood vessels were imaged in the resting-chondrocyte zone. (**b–e**) Higher magnification images of the squares in the preceding panels. (**b**) Higher magnification image of square B in **a**. Hypertrophied resting-chondrocytes (right) surrounded the

blood vessels (left). (**c**) Collage of high magnification images of blood vessel assembly indicated by the square C in **a**. Red blood cells were imaged inside the blood vessel assembly (arrows). Hypertrophied resting chondrocytes were observed in the upper right. (**d**) Blood cells, presumably erythrocytes in the entrance of the narrower blood vessels. (**e**) High magnification image of Figure 8e. Blood cells, presumably erythrocytes aligned in a vestibule of the vessel assembly.

Reference

1. Jing, Y. *et al*. Chondrogenesis and osteogenesis are one continuous developmental and lineage defined biological process. *Sci. Rep.* **7,** 10020; 10.1038/s41598-017-10048-z (2017).
